# Electro-Osmotic Flow and Mass Transfer through a Rough Microchannel with a Modulated Charged Surface

**DOI:** 10.3390/mi15070882

**Published:** 2024-07-04

**Authors:** Yun Qing, Jiaqi Wang, Fengqin Li

**Affiliations:** School of Mathematical Science, Inner Mongolia University, Hohhot 010021, China; 17614817013@163.com (Y.Q.); 32136084@mail.imu.edu.cn (J.W.)

**Keywords:** modulated charged surface, roughness, alternating current electric field, electro-osmotic flow, mass transfer

## Abstract

In this paper, we investigate the electro-osmotic flow (EOF) and mass transfer of a Newtonian fluid propelled by a pressure gradient and alternating current (AC) electric field in a parallel microchannel with sinusoidal roughness and modulated charged surfaces. The two-wall roughness is described by in-phase or out-of-phase sine functions with a small amplitude δ. By employing the method of perturbation expansion, the semi-analytical solutions of the Poisson–Boltzmann (P–B) equation based on the Debye–Hückel approximation and the modified Navier–Stokes (N–S) equation are obtained. The numerical solution of the concentration equation is obtained by the finite difference method. The effects of sinusoidal roughness, modulated charged surface, and the AC electric field on the potential field, velocity field, and concentration field are discussed. Under the influence of the modulated charged surface and sinusoidal roughness, vortices are generated. The velocity oscillates due to the effect of the AC electric field. The results indicate that solute diffusion becomes enhanced when the oscillation Reynolds number is below a specific critical value, and it slows down when the oscillation Reynolds number exceeds this critical value.

## 1. Introduction

In recent years, microfluidic technology has been widely used in biology, medicine, lab-on-a-chip, social production, and other fields due to its advantages, such as low sample consumption, small structure, and portability [1,2,3,4,5]. For example, it can perform fluid transfer, mixing, and other tasks. Therefore, the flow, mixing, and heat transfer of fluids in microfluidic devices have become the focus of research [6,7,8,9,10]. Currently, the flow in a microchannel can be driven by the pressure gradient, centrifugal force, electric field, magnetic field, or a combination of these forces [11,12,13,14,15,16,17,18].

Electro-osmosis has become the preferred method for fabricating microdevices due to its low energy consumption [19,20]. When the electrolyte solution is filled in charged surface microchannels, ions with opposite charges to the wall are attracted to the charged surface, thus forming an electric double layer (EDL) [13,17]. When the electric field is applied to both ends of the microchannel, the ions in the electrical double layer (EDL) begin to move under the influence of the electric field force. Because the fluid is viscous, it flows along with the ions in the microchannel. This flow is called the electro-osmotic flow (EOF) [6]. Chang et al. [21] investigated the electro-osmotic flow in a microchannel with a charge slip on a plate surface. The influence of the thermodiffusive effect on the local Debye length thickness in a purely electro-osmotic flow in a parallel flat plate microchannel was theoretically studied by Hernández et al. [22].

Previous studies have been based on uniform potential. However, due to the limitation of the industrial level, it is not possible to achieve uniform distribution of surface charges [23,24]. Mandal et al. [25] used a modulated charged surface to simulate charge inhomogeneity to study the electro-osmotic flow of superimposed fluids under narrow constraints. Bian and Li [26] consider fluid flow in a microchannel with a modulated charged surface. Akhtar Shehraz et al. [27] used fractional derivatives to study the electro-osmotic flow of Maxwell fluids in microchannels with asymmetric wall potential. Wang and Li [28] studied the EOF and heat transfer in the polyelectrolyte-grafted microchannels with modulated charged surfaces. The above results show that the modulation of charged surfaces can generate eddy currents in the flow field, which have positive effects on fluid mixing, solute diffusion, and heat transfer.

Given that the current manufacturing process is unable to achieve perfect smoothness, there will always be some degree of wall roughness. Sadia Siddiqa [29] conducted a study on the transient analysis of natural convective heat transfer based on vertical wavefronts and obtained the solution to the equation using the coordinate transformation method. Buren et al. [30] analyzed the critical wave value of the influence of wall roughness on velocity and potential distribution using the perturbation expansion method. Li et al. [31] investigated the electromagnetohydrodynamic (EMHD) flow in a three-dimensional corrugated wall microchannel. Chang et al. [32] discussed the impact of sinusoidal roughness on the AC EOF of Maxwell fluids in parallel microchannels.

The diffusion process of solutes is the mass transport due to the inhomogeneity of molecular diffusion and fluid flow velocity. The problem of mass transport in microfluidic systems has important guiding significance in practical applications such as drug delivery or toxin separation in medical care and biological systems [33]. More recently, the applications of the AC electric fields have been extended to electro-osmotic flow with enhanced mass transfer, mixing, and material separation [34,35]. Medina et al. [36] studied the pulsating electro-osmotic flow and solute diffusion in microchannels with wall potential asymmetry. Li and Jian [37] studied the problem of solute diffusion in polyelectrolyte-grafted nanochannels driven by the AC electric field. They discovered that there is a critical oscillation Reynolds number value for solute diffusion driven by the AC electric field. The electromagnetic hydrodynamic flow and mass transfer in curved rectangular microchannels were studied by Liu and Jian [38]. They found that the effective diffusivity increases with the oscillation Reynolds number.

According to the author’s knowledge, currently, no relevant studies have discussed the effects of roughness and modulated charged surface coupling on fluid flow. Therefore, this paper examines the electro-osmotic flow in a microchannel with a rough and modulated charged surface, driven by both the AC electric field and the pressure gradient. The P–B equation based on the Debye–Hückel approximation, and the improved N–S equation are solved using the asymptotic expansion method. The numerical solution of the concentration equation using perturbation expansion is obtained by the finite difference method. Finally, the effects of sinusoidal roughness, modulated charged surface, and the AC electric field on potential, velocity, and mass transport are discussed in detail.

## 2. Mathematics Model

**Figure 1:**
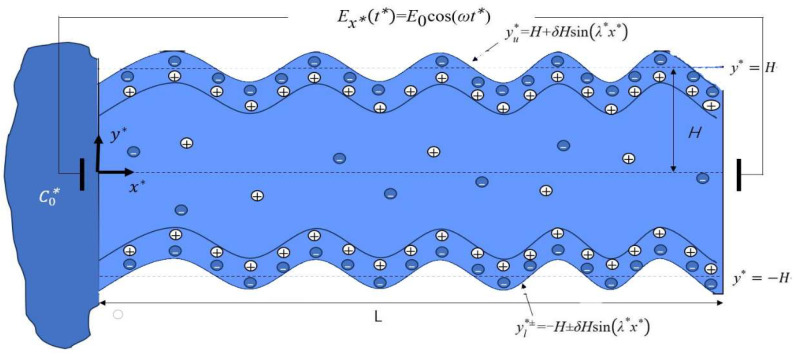
Schematic diagram of mathematical model with roughness microchannel.

In this study, we consider the alternating current electro-osmotic flow and mass transfer of Newtonian fluid in a microchannel with sinusoidal roughness and modulated charged surfaces. Shown as in Figure 1, the length of the mathematical model in the $x^{*}$-axis direction is denoted as *L* (*L* ≫ 2*H*). First, let us consider the low Reynolds number flows in the microchannels. The electric field $E_{x^{*}}$($t^{*}$) is aligned with the $x^{*}$-axis. The fluid is propelled by the AC electric field and pressure gradient. The positions of the upper and lower sinusoidal roughness walls ($y_{u}^{*}$ and $y_{l}^{*}$) can be described by periodic sine waves as follows:

(1a) $$y_{u}^{*}=H+\delta H\sin\left(\lambda^{*}x^{*}\right),$$

(1b) $$y_{l}^{*\pm }=-H\pm \delta H\sin\left(\lambda^{*}x^{*}\right),$$

where λ* is the wave number, and *δ* ≪ 1 is the amplitude. $y_{l}^{*+}=-H+\delta H\sin\left(\lambda^{*}x^{*}\right)$ denotes the in-phase walls, which means that the lower and upper walls are not symmetric. $y_{l}^{*-}=-H\;-\;\delta H\sin\left(\lambda^{*}x^{*}\right)$ denotes the out-of-phase walls, which means that the lower and upper walls are symmetric.

### 2.1. Velocity Distribution

The microchannel walls adopt an asymmetric modulated potential, and the potential distribution function $\psi^{*}\left(x^{*},y^{*}\right)$ of the upper and lower walls is expressed as follows:

(2a) $$\begin{array}{ccc} \psi^{*}\left(x^{*},y^{*}\right)=\xi_{u}^{*}\left[1+\alpha \sin\left(\lambda^{*}x^{*}\right)\right] & \mathrm{at} & y^{*}= \end{array}y_{u}^{*},$$

(2b) $$\begin{array}{ccc} \psi^{*}\left(x^{*},y^{*}\right)=\xi_{l}^{*}\left[1+\beta \sin\left(\lambda^{*}x^{*}\right)\right] & \mathrm{at} & y^{*}= \end{array}y_{l}^{*},$$

where $\xi_{u}^{*}$ and $\xi_{l}^{*}$ are the amplitudes of the upper and lower walls, and α and β are constants. It is assumed that the electrolyte solution fills the microchannel. The potential $\psi^{*}\left(x^{*},y^{*}\right)$ and the net charge density $\rho_{e}^{*}$ can be described by the P–B equation [37]:

(3) $$\nabla^{2}\psi^{*}=-\frac{\rho_{e}^{*}}{\varepsilon },$$

where $\rho_{e}^{*}=$ 2$n_{0}$*ze*sinh(*ze*$\psi^{*}$/${\;k}_{B}T_{a}$) is Boltzmann distribution. The symbol $n_{0}$ represents the concentration of ions in the solution. *z* represents the valence of the ion. *e* is the charge of the electron, $T_{a}$ stands for the absolute temperture, ${\;k}_{B}$ denotes the Boltzmann constant, and *ε* signifies the dielectric constant. By the Debye–Hückel approximation, we obtain the term sinh(*ze*$\psi^{*}$/${\;k}_{B}T_{a}$) ≈ *ze*$\psi^{*}$/${\;k}_{B}T_{a}$. From this we can obtain the approximate P–B equation:

(4) $$\frac{\partial^{2}\psi^{*}}{{\partial x}^{{*}^{2}}}+\frac{\partial^{2}\psi^{*}}{{\partial y}^{{*}^{2}}}=k^{2}\psi^{*},$$

where $\kappa =1/{[({\mathrm{ek}}_{B}T_{a})/(2n_{0}z^{2}e^{2})]}^{1/2}$ denotes the thickness of the EDL, also known as the Debye length. The boundary conditions are (2a) and (2b).

Since both sinusoidal roughness and modulating charge surface effects are taken into account, the velocity is two-dimensional, i.e., $U^{*}=$ ($U^{*}$, $V^{*}$, 0). Then, the continuity equation and the N–S equation can be expressed as [36,37]:

(5) $$\nabla \cdot U^{*}=0,$$

(6) $$\rho (\frac{\partial U^{*}}{\partial t^{*}}+U^{*}\cdot \;\nabla U^{*})=\;-\nabla P^{*}+\mu \nabla^{2}U^{*}+F.$$

There is an external alternating electric field $E_{x^{*}}$(*t*) along the $x^{*}$-axis, and the external force acting on the fluid is *F* $=\rho_{e}^{*}E_{x^{*}}$(t*). Suppose that the AC electric field can be expressed in complex form:

(7) Ex*t*=Re{E0eiωt*},

where *Re*{} represents the real part of the complex number, $E_{0}$ is the amplitude of the applied AC electric field, *i* is the imaginary unit, and *ω* is the oscillating angular frequency of the applied AC electric field. The momentum Equations (5) and (6) are written in a two-dimensional component form. The final Equations (5) and (6) are written as [31]:

(8) $$\frac{\partial U^{*}}{\partial x^{*}}+\frac{\partial V^{*}}{\partial y^{*}}=0,$$

(9) $$\rho (\frac{\partial U^{*}}{\partial t^{*}}+U^{*}\frac{\partial U^{*}}{{\partial x}^{*}}+V^{*}\frac{\partial U^{*}}{{\partial y}^{*}})=-\frac{{\partial P}^{*}}{\partial x^{*}}+\mu (\frac{\partial^{2}U^{*}}{{\partial x}^{{*}^{2}}}+\frac{\partial^{2}U^{*}}{{\partial y}^{{*}^{2}}})+\rho_{e}E_{x}(t^{*}),$$

(10) $$\rho (\frac{\partial V^{*}}{\partial t^{*}}+U^{*}\frac{\partial V^{*}}{{\partial x}^{*}}+V^{*}\frac{\partial V^{*}}{{\partial y}^{*}})=-\frac{{\partial P}^{*}}{\partial y^{*}}+\mu (\frac{\partial^{2}V^{*}}{{\partial x}^{{*}^{2}}}+\frac{\partial^{2}V^{*}}{{\partial y}^{{*}^{2}}}).$$

The boundary conditions for Equations (8)–(10) are as follows:

(11a) $$\begin{array}{ccc} U^{{*}^{\pm }}=0 & \mathrm{at} & y^{*}=y_{u}^{*}\;\mathrm{and}\; \end{array}y^{*}=y_{l}^{*\pm },$$

(11b) $$\begin{array}{ccc} V^{{*}^{\pm }}=0 & \mathrm{at} & y^{*}=y_{u}^{*}\;\mathrm{and}\; \end{array}y^{*}=y_{l}^{*\pm }.$$

Based on the expression of the AC electric field, we assume the complex form of the velocity [37]:

(12) U*x*,y*,t*=Reu*x*,y*eiωt*,V*x*,y*,t*=Rev*x*,y*eiωt*,

where $u^{*}\left(x^{*},y^{*}\right)\;\mathrm{and}\;v^{*}\left(x^{*},\;y^{*}\right)$ are the amplitudes of the periodic velocities in the $x^{*}$ and $y^{*}$ directions, respectively. The following non-dimensional variables are introduced:

(13) $$\left(u,\;v\right)=\frac{\left(u^{*},\;v^{*}\right)}{U_{\mathrm{HS}}},U_{\mathrm{HS}}=\frac{\varepsilon \psi_{s}E_{0}}{\mu },\left(x,\;y\right)=\frac{\left(x^{*},\;y^{*}\right)}{H},\psi =\frac{\psi^{*}}{\psi_{s}},\kappa =\kappa^{*}H,\;\lambda =\lambda^{*}H,\;(\xi_{u},\xi_{l})=\frac{\left(\xi_{u},\;\xi_{l}\right)}{\psi_{s}},\psi_{s}=\frac{k_{B}T_{a}}{\mathrm{ze}},\;P=\frac{P^{*}}{P_{0}},P_{0}=\frac{\mu U_{\mathrm{HS}}}{H},$$

where UHS is the Helmholtz velocity of the Newtonian fluid. The P–B equation, the continuity equation, and the N–S equation can be transformed as:

(14) $$\frac{\partial^{2}\psi }{\partial x^{2}}+\frac{\partial^{2}\psi }{\partial y^{2}}=\kappa^{2}\psi ,$$

(15) $$\frac{\partial u}{\partial x}+\frac{\partial v}{\partial y}=0,$$

(16) −∂P∂x+∂2u∂x2+∂2u∂y2−iReωu+κ2ψ=0,

(17) $$-\frac{\partial P}{\partial y}+\frac{\partial^{2}v}{\partial x^{2}}+\frac{\partial^{2}v}{\partial y^{2}}-{\mathrm{iRe}}_{\omega }v=0,$$

where Reω=ρωH2μ denotes the oscillation Reynolds number and the competition between the characteristic diffusion time and the characteristic time associated with the oscillatory electric field [37]. The non-dimensional boundary conditions for Equations (14)–(17) are

(18a) $$\begin{array}{ccc} \psi (x,\;y)=\xi_{u}[1+\alpha \sin(\lambda x)] & \mathrm{at} & y=y_{u}=1+\delta \sin(\lambda x) \end{array},$$

(18b) $$\begin{array}{ccc} \psi (x,\;y)=\xi_{l}[1+\beta \sin(\lambda x)] & \mathrm{at} & y=y_{l}^{\pm }=-1\pm \delta \sin(\lambda x) \end{array},$$

(18c) $$\begin{array}{ccc} {u(x,\;y)}^{\pm }=0 & \mathrm{at} & \;y=y_{u}\;\mathrm{and} \end{array}\;y=y_{l}^{\pm },$$

(18d) $$\begin{array}{ccc} {v(x,\;y)}^{\pm }=0 & \mathrm{at} & \;y=y_{u}\;\mathrm{and} \end{array}\;y=y_{l}^{\pm },$$

where boundary conditions (18c,18d) illustrate the no-slip and non-permeable boundary conditions.

The asymptotic method applies to problems in which the geometry of the microchannel is complicated and irregular in the sense that the boundaries do not correspond to coordinate surfaces of any known analytic coordinate system, but are nevertheless “near” to such coordinate surfaces [39]. For small values of *δ* ≪ 1, function Ƒ (could be *u*, *v*, *ψ*, *P*, φ, *C*) can be expanded asymptotically by means of perturbation [30,31]:

(19) $$Ƒ={Ƒ}_{0}+\delta {Ƒ}_{1}+\delta^{2}{Ƒ}_{2}+ο\left(\delta^{2}\right).$$

Equation (19) is expanded up to the second-order terms. For the asymptotic expansion of the boundary condition (18a–18d), the Taylor expansion at the mean wall position *y* = 1 and *y* = −1 yields the following:

(20a) $${\psi |}_{y=\xi_{u}[1+\alpha \sin\left(\lambda x\right)]}={\psi |}_{y=1}+\delta \sin\left(\lambda x\right){\frac{\partial \psi }{\partial y}|}_{y=1}+\frac{1}{2}\delta^{2}\sin^{2}(\lambda x){\frac{\partial^{2}\psi }{\partial y^{2}}|}_{y=1}+o(\delta^{2}),$$

(20b) $${\psi |}_{y=\xi_{l}[1+\beta \sin\left(\lambda x\right)]}={\psi |}_{y=-1}\pm \delta \sin\left(\lambda x\right){\frac{\partial \psi }{\partial y}|}_{y=-1}\pm \frac{1}{2}\delta^{2}\sin^{2}(\lambda x){\frac{\partial^{2}\psi }{\partial y^{2}}|}_{y=-1}+o(\delta^{2}),$$

(20c) $$0={u|}_{y=\xi_{u}[1+\alpha \sin\left(\lambda x\right)]}={u|}_{y=1}+\delta \sin(\lambda x){\frac{\partial u}{\partial y}|}_{y=1}+\frac{1}{2}\delta^{2}\sin^{2}(\lambda x){\frac{\partial^{2}u}{\partial y^{2}}|}_{y=1}+ο(\delta^{2}),$$

(20d) $$0={u|}_{y=\xi_{l}[1+\beta \sin\left(\lambda x\right)]}={u|}_{y=-1}\pm \delta \sin\left(\lambda x\right){\frac{\partial u}{\partial y}|}_{y=-1}\pm \frac{1}{2}\delta^{2}\sin^{2}(\lambda x){\frac{\partial^{2}u}{\partial y^{2}}|}_{y=-1}+o(\delta^{2}),$$

(20e) $$0={v|}_{y=\xi_{u}[1+\alpha \sin\left(\lambda x\right)]}={v|}_{y=1}+\delta \sin(\lambda x){\frac{\partial v}{\partial y}|}_{y=1}+\frac{1}{2}\delta^{2}\sin^{2}(\lambda x){\frac{\partial^{2}v}{\partial y^{2}}|}_{y=1}+ο(\delta^{2}),$$

(20f) $$0={v|}_{y=\xi_{l}[1+\beta \sin\left(\lambda x\right)]}={v|}_{y=-1}\pm \delta \sin\left(\lambda x\right){\frac{\partial v}{\partial y}|}_{y=-1}\pm \frac{1}{2}\delta^{2}\sin^{2}(\lambda x){\frac{\partial^{2}v}{\partial y^{2}}|}_{y=-1}+o(\delta^{2}),$$

The asymptotic expansion equation of (*u*, *v*, *ψ*, *P*) is substituted into Equations (14)–(17) and (20a–20f) to obtain the zero-order, first-order, and second-order equations, as well as the boundary conditions.

#### 2.1.1. The Zeroth-Order Equations

The zeroth-order equations are as follows:

(21) $$\frac{\partial^{2}\psi_{0}}{\partial x^{2}}+\frac{\partial^{2}\psi_{0}}{\partial y^{2}}=\kappa^{2}\psi_{0},$$

(22) $$\frac{\partial u_{0}}{\partial x}+\frac{\partial v_{0}}{\partial y}=0,$$

(23) −∂P0∂x+∂2u0∂x2+∂2u0∂y2−iReωu0+κ2ψ0=0,

(24) $$-\frac{\partial P_{0}}{\partial y}+\frac{\partial^{2}v_{0}}{\partial x^{2}}+\frac{\partial^{2}v_{0}}{\partial y^{2}}-{\mathrm{iRe}}_{\omega }v_{0}=0.$$

The zeroth-order boundary conditions are as follows:

(25a) $$\begin{array}{ccc} \psi_{0}\left(x,\;y\right)=\xi_{u}\left[1+\alpha \sin(\lambda x\right)] & \mathrm{at} & y=1, \end{array}$$

(25b) $$\begin{array}{ccc} \psi_{0}\left(x,\;y\right)=\xi_{l}\left[1+\beta \sin(\lambda x\right)] & \mathrm{at} & y=-1 \end{array},$$

(25c) $$\begin{array}{ccc} u_{0}\left(x,\;y\right)=0 & \mathrm{at} & y=1\;\mathrm{and}\; \end{array}y=-1,$$

(25d) $$\begin{array}{ccc} v_{0}\left(x,\;y\right)=0 & \mathrm{at} & y=1\;\mathrm{and}\; \end{array}y=-1.$$

The Equation (21) and boundary conditions (25a,25b) are used to solve the zeroth-order potential solution. Based on the superposition principle, the solution of the potential is as follows:

(26) $$\psi_{0}=f_{1}\left(y\right)+f_{2}\left(y\right)\sin(\lambda x).$$

The expression of coefficient is in Appendix A. In the following calculation, in order to solve the two-dimensional velocity, we introduce the zeroth-order stream function $\varphi_{0}$ according to the zeroth-order continuity equation:

(27) $$u_{0}=\frac{\partial \varphi_{0}}{\partial y},\;v_{0}=-\frac{\partial \varphi_{0}}{\partial x}.$$

By substituting Equation (27) into Equations (22)–(24) of zero-order, the pressure $P_{0}$ is eliminated, and the equation of $\varphi_{0}$ is obtained as follows:

(28) $$2\frac{\partial^{4}\varphi_{0}}{\partial x^{2}\partial y^{2}}+\frac{\partial^{4}\varphi_{0}}{\partial y^{4}}+\frac{\partial^{4}\varphi_{0}}{\partial x^{4}}+\kappa^{2}\frac{\partial \psi_{0}}{\partial y}\;-{\mathrm{iRe}}_{\omega }\left(\frac{\partial^{2}\varphi_{0}}{\partial y^{2}}+\frac{\partial^{2}\varphi_{0}}{\partial x^{2}}\right)=0.$$

The solution of the zeroth-order stream function has the form as follows:

(29) $$\varphi_{0}=g_{1}\left(y\right)+g_{2}\left(y\right)\sin(\lambda x).$$

The expression of coefficient is in Appendix A. Then, the zeroth-order velocity $u_{0}$, $v_{0}$ can be obtained through Equation (31).

#### 2.1.2. The First-Order Equations

The first-order equations are obtained as follows:

(30) $$\frac{\partial^{2}\psi_{1}}{\partial x^{2}}+\frac{\partial^{2}\psi_{1}}{\partial y^{2}}=\kappa^{2}\psi_{1},$$

(31) $$\frac{\partial u_{1}}{\partial x}+\frac{\partial v_{1}}{\partial y}=0,$$

(32) −∂P1∂x+∂2u1∂x2+∂2u1∂y2 −iReωu1+κ2ψ1=0,

(33) $$-\frac{\partial P_{1}}{\partial y}+\frac{\partial^{2}v_{1}}{\partial x^{2}}+\frac{\partial^{2}v_{1}}{\partial y^{2}}\;-{\mathrm{iRe}}_{\omega }v_{1}=0.$$

The first-order boundary conditions are as follows:

(34a) $$\begin{array}{ccc} \psi_{1}{|}_{y=1}=-\sin\left(\lambda x\right){\left(\frac{\partial \psi_{0}}{\partial y}\right)}_{y=1} & , & \psi_{1}{|}_{y=-1}=\mp \sin\left(\lambda x\right){\left(\frac{\partial \psi_{0}}{\partial y}\right)}_{y=-1} \end{array},$$

(34b) $$\begin{array}{ccc} u_{1}{|}_{y=1}=-\sin(\lambda x){\left(\frac{\partial u_{0}}{\partial y}\right)}_{y=1} & , & u_{1}^{\pm }{|}_{y=-1}=\mp \sin(\lambda x){\left(\frac{\partial u_{0}}{\partial y}\right)}_{y=-1} \end{array},$$

(34c) $$\begin{array}{ccc} v_{1}{|}_{y=1}=-\sin(\lambda x){\left(\frac{\partial v_{0}}{\partial y}\right)}_{y=1} & , & v_{1}^{\pm }{|}_{y=-1}=\mp \sin(\lambda x){\left(\frac{\partial v_{0}}{\partial y}\right)}_{y=-1} \end{array}.$$

According to the superposition principle and boundary condition (34a), the first-order potential solution has the following form:

(35) $$\psi_{1}^{\pm }=f_{3}^{\;\pm }\left(y\right)+f_{4}^{\;\pm }\left(y\right)\sin\left(\lambda x\right)+f_{5}^{\;\pm }\left(y\right)\cos\left(2\lambda x\right).$$

The expression of coefficient is in Appendix A. Introducing first-order stream function $\varphi_{1}$ into Equations (31)–(33), the pressure $P_{1}$ can be eliminated, yielding the following equation:

(36) $$2\frac{\partial^{4}\varphi_{1}}{\partial x^{2}\partial y^{2}}+\frac{\partial^{4}\varphi_{1}}{\partial y^{4}}+\frac{\partial^{4}\varphi_{1}}{\partial x^{4}}+\kappa^{2}\frac{\partial \psi_{1}}{\partial y}-{\mathrm{iRe}}_{\omega }\left(\frac{\partial^{2}\varphi_{1}}{\partial y^{2}}+\frac{\partial^{2}\varphi_{1}}{\partial x^{2}}\right)=0.$$

The analytical solution of the first-order stream function $\varphi_{1}$ is in the following form:

(37) $$\varphi_{1}=g_{3}\left(y\right)+g_{4}\left(y\right)\sin(\lambda x)+g_{5}^{\pm }\left(y\right)\cos\left(2\lambda x\right).$$

The expression of coefficient is in Appendix A. Then, the first-order velocity $u_{1}$, $v_{1}$ can be obtained.

#### 2.1.3. The Second-Order Equations

As in the above process, the second-order equations are obtained as follows:

(38) $$\frac{\partial^{2}\psi_{2}}{\partial x^{2}}+\frac{\partial^{2}\psi_{2}}{\partial y^{2}}=\kappa^{2}\psi_{2},$$

(39) $$\frac{\partial u_{2}}{\partial x}+\frac{\partial v_{2}}{\partial y}=0,$$

(40) −∂P2∂x+∂2u2∂x2+∂2u2∂y2−iReωu2+κ2ψ2=0,

(41) $$-\frac{\partial P_{2}}{\partial y}+\frac{\partial^{2}v_{2}}{\partial x^{2}}+\frac{\partial^{2}v_{2}}{\partial y^{2}}-{\mathrm{iRe}}_{\omega }v_{2}=0.$$

The second-order boundary conditions are as follows:

(42a) $$\psi_{2}{|}_{y=1}=-\sin\left(\lambda x\right){\left(\frac{\partial \psi_{1}}{\partial y}\right)}_{y=1}-\frac{1}{2}\sin^{2}\left(\lambda x\right){\left(\frac{\partial^{2}\psi_{0}}{\partial y^{2}}\right)}_{y=1},$$

(42b) $$\psi_{2}^{\pm }{|}_{y=-1}=\mp \sin\left(\lambda x\right){\left(\frac{\partial \psi_{1}}{\partial y}\right)}_{y=-1}\mp \frac{1}{2}\sin^{2}\left(\lambda x\right){\left(\frac{\partial^{2}\psi_{0}}{\partial y^{2}}\right)}_{y=-1},$$

(42c) $$u_{2}{|}_{y=1}=-\sin\left(\lambda x\right){\left(\frac{\partial u_{1}}{\partial y}\right)}_{y=1}-\frac{1}{2}\sin^{2}\left(\lambda x\right){\left(\frac{\partial^{2}u_{0}}{\partial y^{2}}\right)}_{y=1},$$

(42d) $$u_{2}^{\pm }{|}_{y=-1}=\mp \sin(\lambda x){\left(\frac{\partial u_{1}}{\partial y}\right)}_{y=-1}\mp \frac{1}{2}\sin^{2}(\lambda x){\left(\frac{\partial^{2}u_{0}}{\partial y^{2}}\right)}_{y=-1},$$

(42e) $$v_{2}{|}_{y=1}=-\sin\left(\lambda x\right){\left(\frac{\partial v_{1}}{\partial y}\right)}_{y=1}-\frac{1}{2}\sin^{2}\left(\lambda x\right){\left(\frac{\partial^{2}v_{0}}{\partial y^{2}}\right)}_{y=1},$$

(42f) $$v_{2}^{\pm }{|}_{y=-1}=\mp \sin\left(\lambda x\right){\left(\frac{\partial v_{1}}{\partial y}\right)}_{y=-1}\mp \frac{1}{2}\sin^{2}\left(\lambda x\right){\left(\frac{\partial^{2}v_{0}}{\partial y^{2}}\right)}_{y=-1}.$$

According to the superposition principle and considering Equations (38) and the boundary conditions (42a,42b), the second-order potential solution has the following form:

(43) $$\psi_{2}^{\pm }=f_{6}^{\;\pm }\left(y\right)+f_{7}^{\;\pm }\left(y\right)\sin\left(\lambda x\right)+f_{8}^{\;\pm }\left(y\right)\cos\left(2\lambda x\right)+f_{9}^{\;\pm }\left(y\right)\sin\left(3\lambda x\right).$$

The expression of coefficient is in Appendix A. Introducing first-order stream function $\varphi_{2}$ into Equations (39)–(41), the pressure $P_{2}$ can be eliminated, yielding the following equation:

(44) $$2\frac{\partial^{4}\varphi_{2}}{\partial x^{2}\partial y^{2}}+\frac{\partial^{4}\varphi_{2}}{\partial y^{4}}+\frac{\partial^{4}\varphi_{2}}{\partial x^{4}}+\kappa^{2}\frac{\partial \psi_{2}}{\partial y}-{\mathrm{iRe}}_{\omega }\left(\frac{\partial^{2}\varphi_{2}}{\partial y^{2}}+\frac{\partial^{2}\varphi_{2}}{\partial x^{2}}\right)=0.$$

The analytical solution of the first-order stream function $\varphi_{1}$ is in the following form:

(45) $$\varphi_{2}=g_{6}^{\pm }\left(y\right)+g_{7}^{\pm }\left(y\right)\sin\left(\lambda x\right)+g_{8}^{\pm }\left(y\right)\cos\left(2\lambda x\right)+g_{9}^{\pm }\left(y\right)\sin\left(3\lambda x\right).$$

The expression of coefficient is in Appendix A. Then, the second-order velocity $u_{2}$, $v_{2}$ can be obtained.

From Section 2.1.1, Section 2.1.2 and Section 2.1.3, we can obtain the semi-analytical solution of the velocity of the form as follows:

(46) U(x, y, t)=Re(u0x,y+δu1x,y+δ2u2x,y)eiωt,V(x, y, t)=Re(v0x,y+δv1x,y+δ2v2x,y)eiωt.

### 2.2. Concentration Distribution

In this study, we assume that a container with a constant concentration $C_{0}^{*}$ of substance is placed at one end of the microchannel. As stated in Section 3.1, the velocity is two-dimensional; therefore, solute diffusion in a microchannel can be described by a two-dimensional unsteady convection–diffusion equation [36,37,38]:

(47) $$\frac{\partial C^{*}}{\partial t^{*}}+U^{*}\frac{\partial C^{*}}{{\partial x}^{*}}+V^{*}\frac{\partial C^{*}}{{\partial y}^{*}}=D\left(\frac{\partial^{2}C^{*}}{{\partial x}^{{*}^{2}}}+\frac{\partial^{2}C^{*}}{{\partial y}^{{*}^{2}}}\right),$$

where $C^{*}(x^{*},\;y^{*},\;t*)$ is the concentration and *D* is the diffusion. The boundary conditions are as follows:

(48a) $$C^{*}(0,{\;y}^{*},t^{*})=C_{0},$$

(48b) $$C^{*}\left(x^{*},\;y^{*},\;0\right)=0,\;(x^{*}>0)$$

(48c) $$\begin{array}{cc} \frac{\partial C^{*}(x^{*},\;y^{*},\;t^{*})}{\partial y^{*}}=0 & \begin{array}{cc} \mathrm{at} & y^{*}=y_{u}^{*} \end{array} \end{array}\mathrm{and}\;y^{*}=y_{l}^{*\pm }.$$

Equation (48a) indicates that the concentration at the initial position of the microchannel is a constant $C_{0}^{*}$ at any time. Equation (48b) indicates that the concentration in the channel is zero at the initial time. Equation (48c) is the non-permeable boundary condition. The unsteady convection–diffusion equation and the boundary conditions are transformed into nondimensional forms by employing *H/*$U_{\mathrm{HS}}$ and $C_{0}^{*}$ as time and concentration scale ($t=\frac{t^{*}}{t_{0}},t_{0}=\frac{H}{U_{\mathrm{HS}}},C=\frac{C^{*}}{C_{0}})$, respectively. The dimensionless concentration C(x, y, t) is governed by the following:

(49) $$\frac{\partial C}{\partial t}+U\frac{\partial C}{\partial x}+V\frac{\partial C}{\partial y}=\frac{1}{\mathrm{Pe}}\left(\frac{\partial^{2}C}{{\partial x}^{2}}+\frac{\partial^{2}C}{{\partial y}^{2}}\right),$$

where *Pe*$=\frac{HU_{\mathrm{HS}}}{D}$ denotes the Péclet number, and its physical meaning is the ratio of the rate of convection to the rate of diffusion with the following nondimensional boundary conditions:

(50a) C(0,y, t)=1, C(x, y, 0)=0, (x >0)

(50b) $$\begin{array}{cc} \frac{\partial C(x,y,\;t)}{\partial y}=0 & \begin{array}{cc} \mathrm{at} & y=y_{u}\;\mathrm{and}\;y=y_{l}^{\pm }\; \end{array}\; \end{array}$$

The dimensionless variable C can be expanded asymptotically by utilizing perturbation:

(51) $$C(x,\;y,\;t)=C_{0}(x,\;y,\;t)+\delta C_{1}(x,\;y,\;t)+\delta^{2}C_{2}(x,\;y,\;t)+o(\delta^{2}).$$

From Equations (49)–(51), the zeroth, first, and second order equations, as well as the boundary condition, are obtained.

(52) $$\delta^{0}:\;\;\;\;\frac{\partial C_{0}}{\partial t}+U_{0}\frac{\partial C_{0}}{\partial x}+V_{0}\frac{\partial C_{0}}{\partial y}=\frac{1}{\mathrm{Pe}}\left(\frac{\partial^{2}C_{0}}{{\partial x}^{2}}+\frac{\partial^{2}C_{0}}{{\partial y}^{2}}\right),\;C_{0}\left(0,y,t\right)=1,\;C_{0}\left(x,\;y,0\right)=0,\;(x\;>0)\;\frac{\partial C_{0}(x,\;1,\;t)}{\partial y}=\frac{\partial C_{0}(x,-1,\;t)}{\partial y}=0$$

where U0x, y, t=Reu0x, yeiωt, V0x, y, t=Rev0x, yeiωt.

(53) $$\delta^{1}:\;\;\;\;\frac{\partial C_{1}}{\partial t}+U_{0}\frac{\partial C_{1}}{\partial x}+V_{0}\frac{\partial C_{1}}{\partial y}+U_{1}\frac{\partial C_{0}}{\partial x}+V_{1}\frac{\partial C_{0}}{\partial y}=\frac{1}{\mathrm{Pe}}\left(\frac{\partial^{2}C_{1}}{{\partial x}^{2}}+\frac{\partial^{2}C_{1}}{{\partial y}^{2}}\right),\;C_{1}\left(0,y,\;t\right)=1,\;C_{1}\left(x,\;y,0\right)=0,\;(x\;>0)\;\frac{\partial C_{1}(x,1,\;t)}{\partial y}=-\sin\left(\lambda x\right){\left(\frac{\partial^{2}C_{0}}{\partial y^{2}}\right)}_{y=1}\frac{\partial C_{1}(x,-1,\;t)}{\partial y}=\mp \sin\left(\lambda x\right){\left(\frac{\partial^{2}C_{0}}{\partial y^{2}}\right)}_{y=-1}$$

where U1x, y, t=Reu1x, yeiωt, V1x, y, t=Rev1x, yeiωt.

(54) $$\delta^{2}:\;\;\;\;\frac{\partial C_{2}}{\partial t}+U_{0}\frac{\partial C_{2}}{\partial x}+V_{0}\frac{\partial C_{2}}{\partial y}+U_{1}\frac{\partial C_{1}}{\partial x}+V_{1}\frac{\partial C_{1}}{\partial y}+U_{2}\frac{\partial C_{0}}{\partial x}+V_{2}\frac{\partial C_{0}}{\partial y}=\frac{1}{\mathrm{Pe}}\left(\frac{\partial^{2}C_{2}}{{\partial x}^{2}}+\frac{\partial^{2}C_{2}}{{\partial y}^{2}}\right),\;C_{2}\left(0,y,\;t\right)=1,\;C_{2}\left(x,\;y,0\right)=0,\;(x\;>0)\;\frac{\partial C_{2}(x,\;1,\;t)}{\partial y}=-\sin\left(\lambda x\right){\left(\frac{\partial^{2}C_{1}}{\partial y^{2}}\right)}_{y=1}-\frac{1}{2}{sin}^{2}\left(\lambda x\right){\left(\frac{\partial^{3}C_{0}}{\partial y^{3}}\right)}_{y=1}\;\frac{\partial C_{2}(x,\;-1,\;t)}{\partial y}=\mp \sin\left(\lambda x\right){\left(\frac{\partial^{2}C_{1}}{\partial y^{2}}\right)}_{y=-1}\mp \frac{1}{2}{sin}^{2}\left(\lambda x\right){\left(\frac{\partial^{3}C_{0}}{\partial y^{3}}\right)}_{y=-1}$$

where U2x, y, t=Reu2x, yeiωt, V2x, y, t=Rev2x, yeiωt.

The analytical solution of Equations (52)–(54) is still difficult to obtain. Therefore, to obtain the numerical solution of the equation, the finite difference method (FDM) [40] is utilized in this study. The detailed finite difference scheme and algorithm can be found in Appendix B.

## 3. Results and Discussion

The diffusion of solutes at one end of the microchannel, the microchannel with the rough modulated charged surface driven by an applied electric field, is studied. Some dimensionless parameters play an important role in the fluid motion in the microchannel, such as the oscillation Reynolds number Reω, the roughness amplitude *δ*, the modulation parameters *α*, *β*, and so on. Based on the previous studies, the physical parameters are as follows [32,36]: the half of the microchannel *H* = 100 µm, the density of the fluid *ρ* = 10^3^ kg·m^−3^, and the dynamic viscosity *µ* = 10^−3^ kgm^−1^s^−1^. At the same time, the applied electric field frequency ranges from 0 to 1.6 kHz, and the variation range of the corresponding angular frequency *ω* is from 0 to 10^4^ s^−1^. Therefore, the oscillation Reynolds number Reω could take a value between 0 and 100. In the following discussion about the upper and lower modulated charged surfaces, the ranges of the amplitudes ($\xi_{u}$ and $\xi_{l}$), constants (*α* and *β*), and mode frequencies (*λ*) are 0~0.2, 0~6, and 0~4 respectively. The molecular diffusion coefficient D varies from O(10^−8^)m^2^ s^−1^ to O(10^−9^)m^2^ s^−1^ [36]. The Péclet number *Pe* varies from 0.3 to 1, and the range of *δ* is 0 ≤ δ ≤ 0.1. The initial concentration $C_{0}=$ 10^3^ mol·m^−3^ [41].

### 3.1. Electric Potential Field

**Figure 2:**
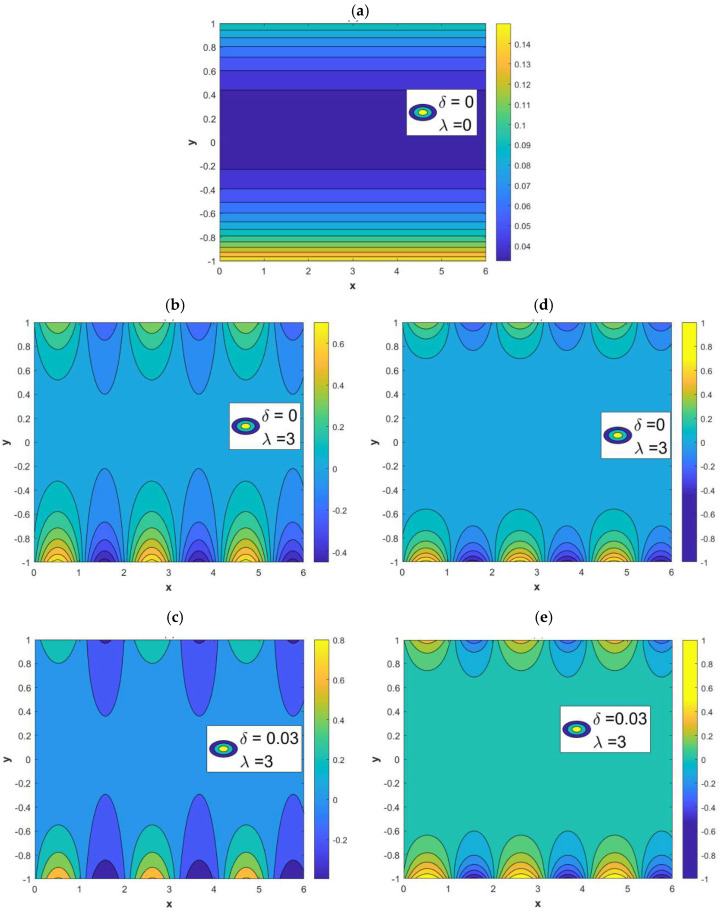
The potential distribution varies with the different variables *δ* and *λ* (*κ* = 2, $\xi_{u}=$ 0.1, $\xi_{l}=$ 0.15, *α* = 3, *β* = 4). (**a**) *δ* = 0, *λ* = 0, non-roughness. (**b**) *δ* = 0, *λ* = 3, in phase. (**c**) *δ* = 0.03, *λ* = 3, in-phase. (**d**) *δ* = 0, *λ* = 3, out-of-phase. (**e**) *δ* = 0.03, *λ* = 3, out-of-phase.

Figure 2 illustrates the impact of sinusoidal roughness and modulated charged surfaces on potential fields. When *λ* = 0 and *δ* = 0 in Figure 2a, the wall represents a smooth parallel plate microchannel and the potential is uniform. At this time, the lines of potential near the wall are denser, indicating a higher potential intensity. It can also be seen from Figure 2a that the potential distribution is not uniform, which is caused by the difference between $\xi_{u}$ and $\xi_{l}$; that is, the two walls potentials are not symmetrical. Figure 2b,c represent potential fields on in-phase walls, and Figure 2d,e represent potential fields on out-of-phase walls. By comparing Figure 2b–e with Figure 2a, it can be observed that the potential distribution on the wall is non-uniform because of the impact of the modulated charged surface and roughness. Then, the potential distribution on the walls will cause the potential inside the microchannel to be uneven.

**Figure 3:**
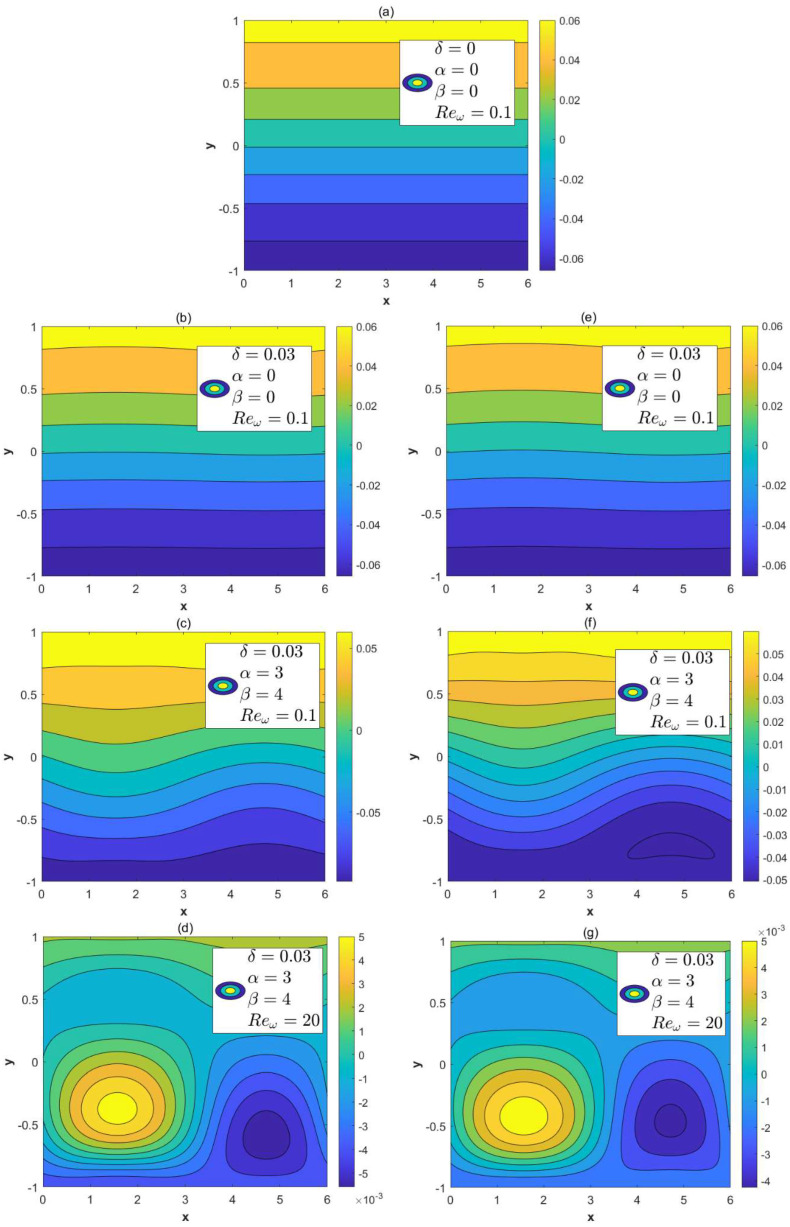
Distributions of streamlines for different parameters Reω, *δ*, *α* and *β* (*λ* = 1, *κ* = 2, $\xi_{u}=$ 0.1, $\xi_{l}=$ 0.15). (**a**) *δ* = 0, *α* = *β* = 0, Reω= 0.1, non-roughness. (**b**) *δ* = 0.03, *α* = *β* = 0, Reω= 0.1, in-phase. (**c**) *δ* = 0.03, *α* = 3, *β* = 4, Reω= 0.1, in-phase. (**d**) *δ* = 0.03, *α* = 3, *β* = 4, Reω= 20, in-phase. (**e**) *δ* = 0.03, *α* = 0, *β* = 0, Reω= 0.1, out-of-phase. (**f**) *δ* = 0.03, *α* = 3, *β* = 4, Reω= 0.1, out-of-phase. (**g**) *δ* = 0.03, *α* = 3, *β* = 4, Reω= 20, out-of-phase.

Figure 3 shows the distribution of streamlines with the sinusoidal roughness amplitude δ, modulated parameters *α*, *β*, and Reω under the conditions of *λ* = 1, *κ* = 2, $\xi_{u}=$ 0.1, $\xi_{l}=$ 0.15. Figure 3a depicts streamlines in the non-rough microchannels with asymmetric wall potentials. The flow at this point is un-directional. Figure 3b–d describe streamlines of the AC EOF for the in-phase walls, while Figure 3e–g depict streamlines of the AC EOF for the out-of-phase walls. Physically, the roughness and modulated charged surfaces change the ion distribution in the electrolyte solution in the microchannel, which affects the distribution of the potential in the EDL. Therefore, it will generate an influence on the flow of the fluid. After adding the effect of the oscillation Reynolds number, the circulation flow is enhanced.

**Figure 4:**
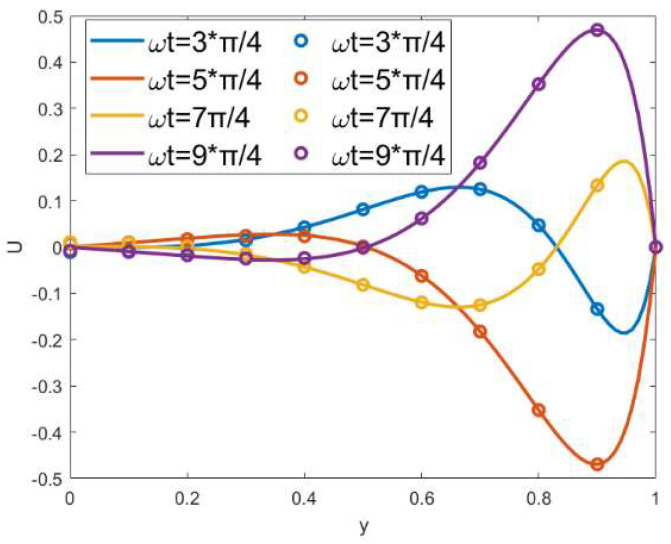
Comparison of the velocity between the results of Medina et al. (solid lines) and semi-solutions (circles) of the present study for different times (t=3π/4, 5π/4, 7π/4, 9π/4), when *α* = *β* = 0, *δ* = 0, *κ* = 20, $\xi_{u}=\xi_{l}=$ 1, *λ* = 1.

When *α* = *β* = 0, *δ* = 0, *κ* = 20, $\xi_{u}=\xi_{l}=$ 1, and *λ* = 1, Figure 4 shows a comparison between the experimental results (solid lines) of Medina et al. [36] and the semi-analytic solution (circles) presented in this paper. Four dimensionless transient velocity curves at different times are drawn in the figure, and the roughness and modulated wall potential are not considered in Figure 4. As can be seen from the figure, there is a high consistency between the two curves.

**Figure 5:**
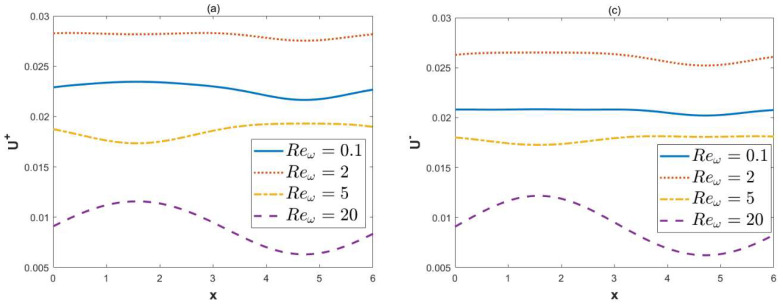

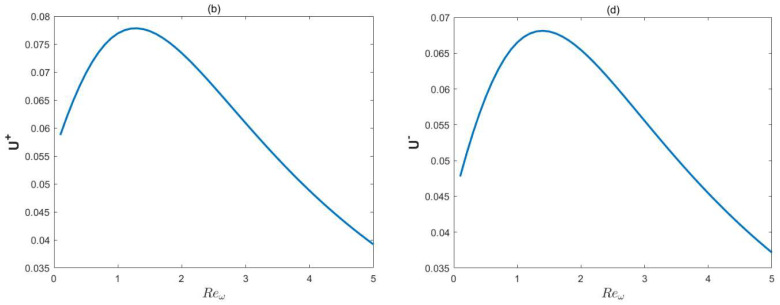
The 2D distributions of dimensionless velocities $U^{\pm }$ with different variables Reω (*κ* = 2, $\xi_{u}=$ 0.1, $\xi_{l}=$ 0.15, *λ* = 1, ω*t* = 1, *δ* = 0.03, *α* = 3, *β* = 4). (**a**) Velocity $U^{+}$ at *y* = 0.8, in-phase. (**b**) Velocity $U^{+}$ at *x* = 2, *y* = 0, in-phase. (**c**) Velocity $U^{-}$ at *y* = 0.8, out-of-phase. (**d**) Velocity $U^{-}$ at *x* = 2, *y* = 0, out-of-phase.

**Figure 6:**
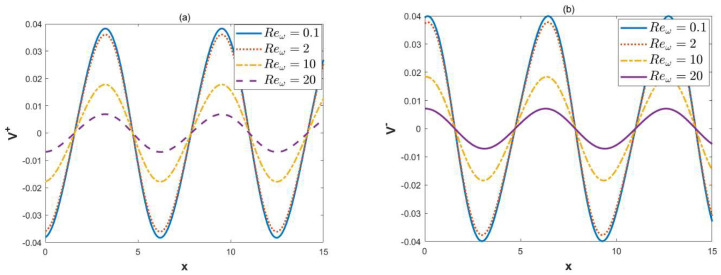
The 2D distributions of dimensionless velocities $V^{\pm }$ with different variables Reω (*κ* = 2, $\xi_{u}=$ 0.1, $\xi_{l}=$ 0.15, *λ* = 1, ω*t* = 0, *δ* = 0.03, *α* = 3, *β* = 4, *y* = 0). (**a**) Velocity $V^{+}$, in-phase. (**b**) Velocity $V^{-}$, out-of-phase.

When *κ* = 2, $\xi_{u}=$0.1, $\xi_{l}=$ 0.15, *λ* = 1, ω*t* = 1, *δ* = 0.03, and *α* = 3, *β* = 4, Figure 5 and Figure 6 describe the variations of dimensionless velocities $U^{\pm }$ and $V^{\pm }$ with variable Reω. Figure 5 illustrates that as the oscillatory Reynolds number increases, the oscillatory nature of the dimensionless velocity $U^{\pm }$ becomes apparent. With the increase in the oscillatory Reynolds number, the amplitude of the velocity first increases and then decreases. Physically, in the case of a high oscillating frequency, the diffusion time is much longer than the oscillating period, which leads to a decrease in the velocity amplitude. Figure 6 shows that as the oscillatory Reynolds number increases, the amplitude of the vertical velocity $V^{\pm }$ decreases. There is no significant difference in the oscillations of vertical velocity for different oscillatory Reynolds numbers.

**Figure 7:**
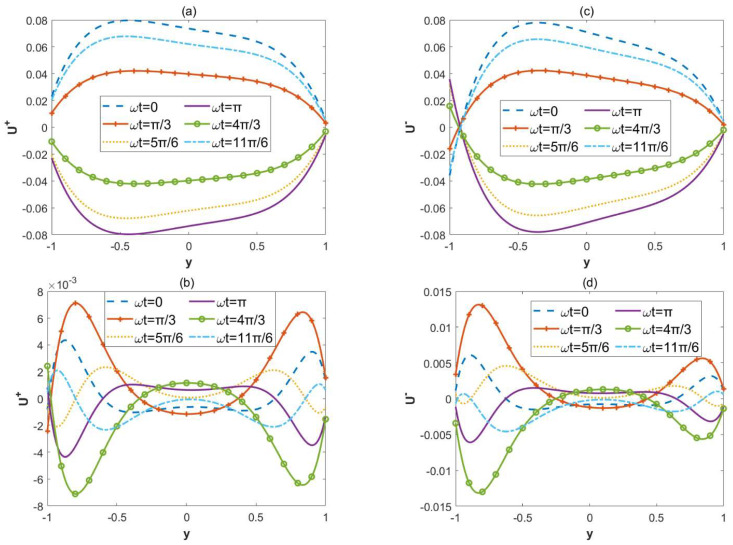
Dimensionless velocity $U^{\pm }$ with different values of ωt(ωt=0, π/3, 5π/6, π, 4π/3, 11π/6) when (*κ* = 2, $\xi_{u}=$ 0.1, $\xi_{l}=$ 0.15, *α* = 3, *β* = 4, *λ* = 1, *δ* = 0.03). (**a**) Reω= 0.1, in-phase. (**b**) Reω= 70, in-phase. (**c**) Reω= 0.1, out-of-phase. (**d**) Reω= 70, out-of-phase.

Figure 7 depicts the change in the transient dimensionless velocity $U^{\pm }$. In Figure 7a,c, Reω= 0.1, and in Figure 7b,d, Reω= 70. With the increase in the oscillatory Reynolds number Reω, the velocity distribution gradually exhibits oscillatory characteristics. The velocity $U^{\pm }$ oscillates in the region close to the wall. And it tends to zero in the region far from the wall at the large oscillatory Reynolds number. This is because, at high electric field frequencies, the diffusion time is greater than the oscillation period.

**Figure 8:**
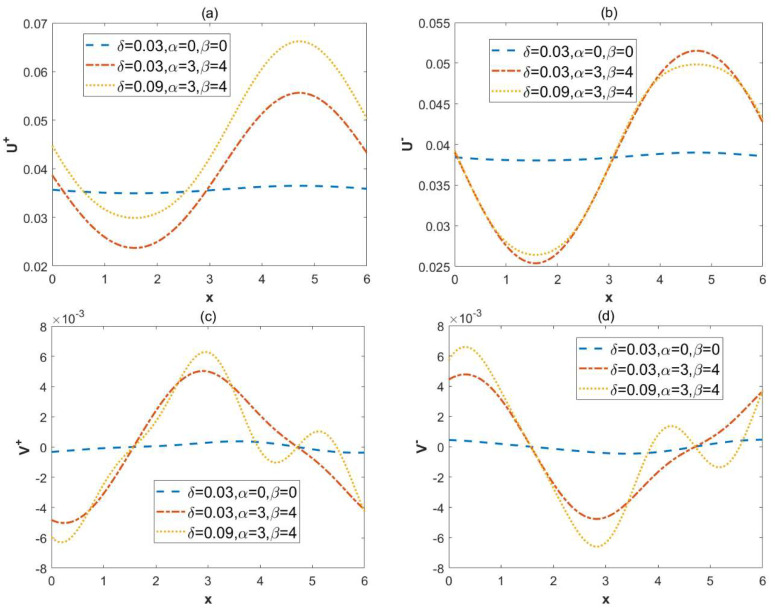
Effect of variables (*α*, *β*, and *δ*) the velocity $U^{\pm }$ and $V^{\pm }$. (*λ* = 1, $\xi_{u}=$ 0.1, $\xi_{l}=$ 0.15, *κ* = 2 Reω= 5 ω*t* = 1). (**a**) the velocity $U^{+}$ at *y* = 0, in-phase. (**b**) the velocity $U^{-}$ at *y* = 0, out-of-phase. (**c**) the velocity $V^{\pm }$ at *y* = 0.8, in-phase. (**d**) the velocity $V^{-}$ at *y* = 0.8, out-of-phase.

Figure 8 illustrates the effect of the charge modulation surface and the sinusoidal roughness on the velocities $U^{\pm }$ and $V^{\pm }$. In Figure 8a,b, the dimensionless velocity $U^{\pm }$ profile is presented, while Figure 8c,d displays the dimensionless velocity $V^{\pm }$ profile. Figure 8a,b indicates that the amplitude of velocity $U^{\pm }$ becomes enhanced with the application of modulation potential. This is because as the amplitude of the modulation potential heightens, the potential in the EDL also increases, leading to a rise in the amplitude of the velocity $U^{\pm }$. When the roughness increases, it can be observed from Figure 8a that the velocity $U^{+}$ becomes incremental. However, Figure 8b shows that the velocity $U^{-}$ slows down with the expansion of the roughness. In Figure 8c,d, it is observed that as the amplitude of the modulation potential increases, the amplitude of the velocity $V^{\pm }$ also increases. The rise of the roughness highlights the oscillation characteristics of the velocity $V^{\pm }$.

### 3.2. Concentration Field

**Figure 9:**
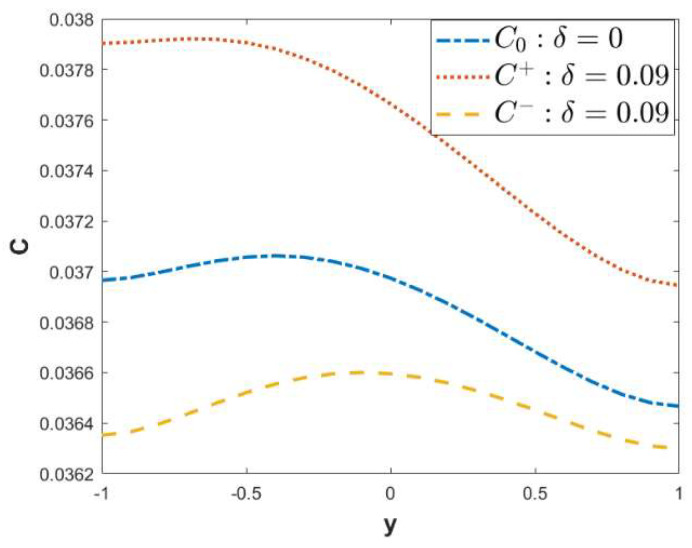
The comparison of concentrations on three different surfaces: non-roughness, in-phase and out-of-phase ($\xi_{u}=$ 0.1, $\xi_{l}=$ 0.15, *α* = 3, *β* = 4, *x* = 3, *λ* = 1, *κ* = 2, ω*t* = 1, Reω= 0.1, *Pe* = 1).

When $\xi_{u}=$ 0.1, $\xi_{l}=$ 0.15, *α* = 3, *β* = 4, *x* = 3, *λ* = 1, *κ* = 2, ω*t* = 1, and ${Re}_{\omega }=$ 0.1, Figure 9 shows the comparison of concentrations at the fixed point *x* = 3 of the microchannel with three different wall surfaces: non-roughness ($C_{0}$), in-phase ($C^{+}$), and out-of-phase ($C^{-}$). The concentration $C^{+}$ is higher than the concentration $C_{0}$ of the non-roughness, which indicates that the solute diffusion rate on the in-phase walls is higher than that on the non-roughness walls. On the contrary, the concentration $C^{-}$ is smaller than the concentration $C_{0}$; that is, the solute diffusivity on the out-of-phase walls is smaller than that of the non-roughness walls. It can be seen from Figure 8a,b that the velocity $U^{+}$ of the in-phase walls will increase with the increase in the roughness. However, the velocity $U^{-}$ of the out-of-phase walls will decrease accordingly. These lead to the difference in the solute diffusion of the three walls. Due to the shape of the out-of-phase walls, the fluid resistance is generated in the microchannel, which hinders the flow of the fluid [31]. Consequently, solute diffusion in the microchannel is reduced.

**Figure 10:**
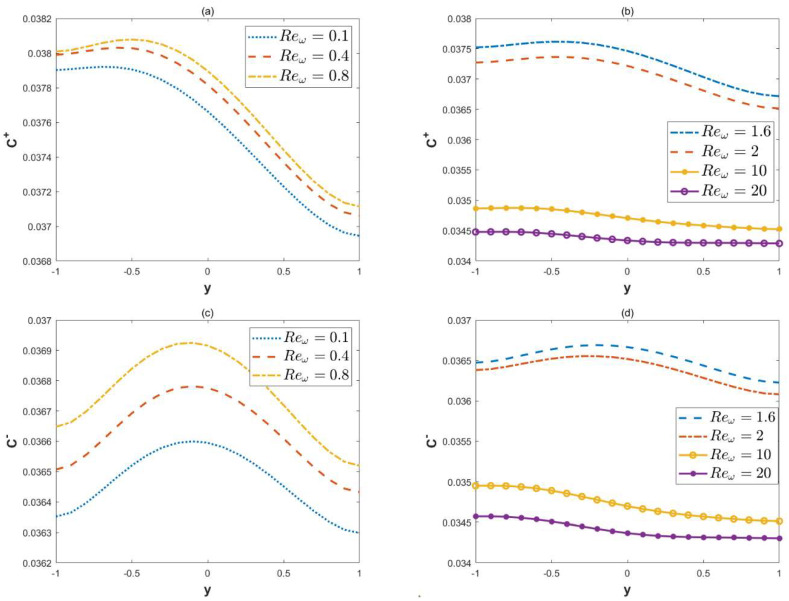
The 2D distributions of dimensionless concentrations $C^{\pm }$ with different variables Reω (*κ* = 2, $\xi_{u}=$ 0.1, $\xi_{l}=$ 0.15, *λ* = 1, ω*t* = 1, *δ* = 0.03, *α* = 3, *β* = 4, *x* = 3, *Pe* = 1). (**a**) Concentrations $C^{+}$, in-phase. (**b**) Concentrations $C^{+}$, in-phase (**c**) Concentrations $C^{-}$, out-of-phase. (**d**) Concentrations $C^{-}$, out-of-phase.

Figure 10 illustrates the changes in the concentration with the oscillation Reynolds number. It can be found that, with the increment of the oscillation Reynolds number, the local concentration $C^{\pm }$ increases when the oscillation Reynolds number is below a certain critical value, and the concentration $C^{\pm }$ will decrease when it exceeds this critical value. The reason is clear: although the large oscillation Reynolds number will improve the oscillation of the velocity, it will also lead to a decrease in the amplitude of the velocity.

**Figure 11:**
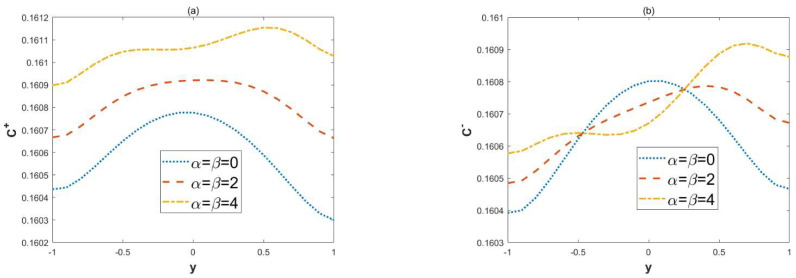
The 2D distributions of dimensionless concentrations $C^{\pm }$ with different variables *α*, *β* (*κ* = 2, $\xi_{u}=$ 0.1, $\xi_{l}=$ 0.1, *λ* = 1, ω*t* = 1, *δ* = 0.03, Reω= 5, *x* = 2, *Pe* = 1). (**a**) Concentrations $C^{+}$, in-phase. (**b**) Concentrations $C^{-}$, out-of-phase.

Figure 11 shows the change in solute diffusions with the modulation charged parameters *α* and *β*. In Figure 11, *α* = *β* = 0 represents the microchannel with no modulated charged surface; i.e., only the effect of the symmetric walls potential is present. When *α* and *β* are not zero, they represent modulated charged surface microchannels. In these cases, the local concentration $C^{+}$ also increases due to the effect of the modulated charged surface. This is because modulating the charged surface increases the amplitude of the velocity, which in turn leads to faster diffusion of the solute. Due to the combined influence of roughness and modulated charged surface, the profile of concentration $C^{-}$ shows an oscillating nature.

**Figure 12:**
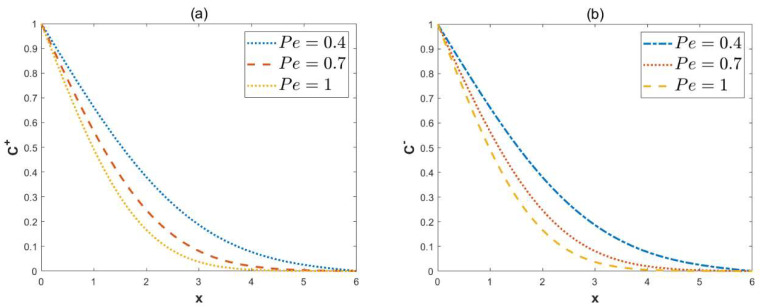
The 2D distributions of dimensionless concentrations $C^{\pm }$ with different variables *Pe* (*κ* = 2, $\xi_{u}=$ 0.1, $\xi_{l}=$ 0.15, *λ* = 1, ω*t* = 1, *δ* = 0.03, Reω= 5, *y* = 0.8, *α* = 3, *β* = 4). (**a**) Concentrations $C^{+}$, in-phase. (**b**) Concentrations $C^{-}$, out-of-phase.

Figure 12 illustrates the change in concentrations with the Péclet number Pe. In Figure 12, the diffusion of solute in the microchannel slows down as the Pe increases. This is because a low Péclet number Pe represents the diffusion term dominant. The flow with a small Reynolds number is considered in this study. Solutes in low Reynolds number flows are mainly affected by diffusion mechanisms. As the Péclet number Pe increases, the restricted diffusion mechanism causes a decrease in the local concentration $C^{\pm }$.

## 4. Conclusions

In this paper, the AC EOF and mass transport in microchannels with modulated charged surfaces and roughness are investigated. The effects of modulating a charged surface, sinusoidal roughness, and AC electric field on electrical potential, velocity, and mass transport are discussed in detail. The variations in potential, velocities, and concentration depend on some dimensionless parameters, such as oscillating Reynolds number (Reω), the roughness parameter (*δ*), the modulated charged surfaces parameter (*α*, *β*), and the Péclet number (*Pe*). By employing a theoretical analysis and a MATLAB R2021b calculation, the following conclusions are achieved:

- When the modulated charge surface parameters (*α*, *β*) increases, the vertical velocity and the circulation flow are generated; the amplitudes of velocities also increase. In these cases, the local concentration of in-phase walls also increase.
- As the roughness parameter *δ* amplifies, the oscillation of the vertical velocity is enhanced. The in-phase walls are conducive to the diffusion of solutes.
- With the increment in the oscillation Reynolds number, the oscillations of the transverse velocity also increase. It can be found that the local concentrations will increase when the oscillation Reynolds number is below a certain critical value.

## Data Availability

The original contributions presented in the study are included in the article, further inquiries can be directed to the corresponding author/s.

## Appendix A. Mathematical Formulas

The zero-order equation correlation coefficients as well as constant terms.

(A1) $$f_{1}\left(y\right)=\frac{\xi_{u}\sin h[\kappa (1+y)]+\xi_{l}\sin h[\kappa (1-\;y)]}{\sin h(2\kappa )}$$

(A2) $$f_{2}\left(y\right)=\frac{\xi_{u}\alpha \sin h[A(1+y)]+\xi_{l}\beta \sin h[A(1\;-\;y)]}{\sin h(2A)}$$

(A3) $$A=\sqrt{\lambda^{2}+\kappa^{2}}$$

(A4) $$g_{1}\left(y\right)=c_{13}e^{\sqrt{{\mathrm{iRe}}_{\omega }}y}+c_{14}e^{-\sqrt{{\mathrm{iRe}}_{\omega }}y}+A_{1}\cos h\left[k\left(1+y\right)\right]+B_{1}\cos h\left[k\left(1-y\right)\right]$$

(A5) $$g_{2}\left(y\right)=c_{21}e^{\lambda y}+c_{22}e^{-\lambda y}+c_{23}e^{\mathrm{Cy}}{+c}_{24}e^{-\mathrm{Cy}}+A_{2}\cos h\left[k\left(1+y\right)\right]+B_{2}\cos h\left[k\left(1-y\right)\right]$$

(A6) $$\begin{array}{ccc} A_{1}=\frac{-k\xi_{u}}{B\sin h\left(2k\right)} & B_{1}=\frac{k\xi_{l}}{B\sin h\left(2k\right)} & B=k^{2}-\;{\mathrm{iRe}}_{\omega } \end{array}$$

(A7) $$\begin{array}{ccc} A_{2}=\frac{-k^{2}\xi_{u}\alpha A}{D_{2}\sin h\left(2A\right)} & B_{2}=\frac{k^{2}\xi_{l}\beta A}{D_{2}\sin h\left(2A\right)} & D_{2}=\frac{1}{\left(A^{2}-\lambda^{2}\right)\left(A^{2}-\left(\lambda^{2}+{\mathrm{iRe}}_{\omega }\right)\right)} \end{array}C=\sqrt{\lambda^{2}+{\mathrm{iRe}}_{\omega }}$$

The correlation coefficient satisfies the following equation $\chi_{i}\gamma_{i}=\;{Г}_{i}$, (i = 1, 2):

(A8) $$\gamma_{1}=\left(\begin{array}{c} c_{13}\\ c_{14} \end{array}\right)$$

(A9) $$\chi_{1}=\left(\begin{array}{cc} \sqrt{{\mathrm{iRe}}_{\omega }}\exp(\sqrt{{\mathrm{iRe}}_{\omega }}) & -\sqrt{{\mathrm{iRe}}_{\omega }}\exp(-\sqrt{{\mathrm{iRe}}_{\omega }})\\ \sqrt{{\mathrm{iRe}}_{\omega }}\exp(-\sqrt{{\mathrm{iRe}}_{\omega }}) & -\sqrt{{\mathrm{iRe}}_{\omega }}\exp(\sqrt{{\mathrm{iRe}}_{\omega }}) \end{array}\right)$$

(A10) $${Г}_{1}=\left(\begin{array}{c} \frac{k^{2}\xi_{u}}{B}\\ \frac{k^{2}\xi_{l}}{B} \end{array}\right)$$

(A11) $$\gamma_{2}=\left(\begin{array}{c} \gamma_{21}\\ \gamma_{22}\\ \gamma_{23}\\ \gamma_{24} \end{array}\right)$$

(A12) $$\chi_{2}=\left(\begin{array}{cccc} \lambda \exp(\lambda ) & -\lambda \exp(-\lambda ) & C\exp(C) & -C\exp(-C)\\ \lambda \exp(-\lambda ) & -\lambda \exp(\lambda ) & C\exp(-C) & -C\exp(C)\\ \exp(\lambda ) & \exp(-\lambda ) & \exp(C) & \exp(-C)\\ \exp(-\lambda ) & \exp(\lambda ) & \exp(-C) & -\exp(C) \end{array}\right)$$

(A13) $$\Gamma_{2}=\left(\begin{array}{c} -A_{2}A\sin h(2A)\\ B_{2}A\sin h(2A)\\ {-A}_{2}\cos h\left(2A\right)-B_{2}\\ {-A}_{2}-B_{2}\cos h\left(2A\right) \end{array}\right)$$

The first-order equation correlation coefficients as well as constant terms.

(A14) $$f_{3}^{\;\pm }\left(y\right)=\frac{-\frac{1}{2}{\left(\frac{\partial f_{2}}{\partial y}\right)}_{y=1}\sin h[\kappa (1+y)]\mp \frac{1}{2}{\left(\frac{\partial f_{2}}{\partial y}\right)}_{y=\;-1}\sin h[\kappa (1-y)]}{\sin h(2\kappa )}$$

(A15) $$f_{4}^{\;\pm }\left(y\right)=\frac{-{\left(\frac{\partial f_{1}}{\partial y}\right)}_{y=1}\sin h[A(1+y)]\mp {\left(\frac{\partial f_{1}}{\partial y}\right)}_{y=\;-1}\sin h[A(1-y)]}{\sin h(2A)}$$

(A16) $$f_{5}^{\;\pm }\left(y\right)=\frac{\frac{1}{2}{\left(\frac{\partial f_{2}}{\partial y}\right)}_{y=1}\sin h[B(1+y)]\pm \frac{1}{2}{\left(\frac{\partial f_{2}}{\partial y}\right)}_{y=\;-1}\sin h[D(1-y)]}{\sin h(2D)}$$

(A17) $$D=\sqrt{(2\lambda {)}^{2}+\kappa^{2}}$$

(A18) $$g_{3}^{\pm }\left(y\right)=c_{33}e^{\sqrt{{\mathrm{iRe}}_{\omega }}y}+c_{34}e^{-\sqrt{{\mathrm{iRe}}_{\omega }}y}+A_{3}^{\pm }\cos h\left[k\left(1+y\right)\right]+B_{3}^{\pm }\cos h\left[k\left(1-y\right)\right]$$

(A19) $$g_{4}^{\pm }\left(y\right)=c_{41}e^{\lambda y}+c_{42}e^{-\lambda y}+c_{43}e^{\mathrm{Cy}}{+c}_{44}e^{-\mathrm{Cy}}+A_{4}^{\pm }\cos h\left[A\left(1+y\right)\right]+B_{4}^{\pm }\cos h\left[A\left(1-y\right)\right]$$

(A20) $$g_{5}^{\pm }\left(y\right)=c_{51}e^{2\lambda y}+c_{52}e^{-2\lambda y}+c_{53}e^{\mathrm{Ey}}{+c}_{54}e^{-\mathrm{Ey}}+A_{5}^{\pm }\cos h\left[D\left(1+y\right)\right]+B_{5}^{\pm }\cos h\left[D\left(1-y\right)\right]$$

(A21) $$\begin{array}{ccc} A_{3}=\frac{-\frac{1}{2}{\left(\frac{\partial f_{2}}{\partial y}\right)}_{y=1}}{B\sin h\left(2k\right)} & B_{3}^{\pm }=\frac{\pm \frac{1}{2}{\left(\frac{\partial f_{2}}{\partial y}\right)}_{y=\;-1}}{B\sin h\left(2k\right)} & E=\sqrt{{4\lambda }^{2}+{\mathrm{iRe}}_{\omega }} \end{array}$$

(A22) $$\begin{array}{ccc} A_{4}=\frac{k^{2}{\left(\frac{\partial f_{1}}{\partial y}\right)}_{y=1}A}{D_{2}\sin h\left(2A\right)} & B_{4}^{\pm }=\frac{\mp k^{2}{\left(\frac{\partial f_{1}}{\partial y}\right)}_{y=\;-1}A}{D_{2}\sin h\left(2A\right)} & D_{2}=\frac{1}{\left(A^{2}-\lambda^{2}\right)\left(A^{2}-\left(\lambda^{2}+{\mathrm{iRe}}_{\omega }\right)\right)} \end{array}$$

(A23) $$\begin{array}{ccc} A_{5}=\frac{-{\frac{1}{2}k}^{2}{\left(\frac{\partial f_{2}}{\partial y}\right)}_{y=1}D}{D_{5}\sin h\left(2A\right)} & B_{5}^{\pm }=\frac{\pm {\frac{1}{2}k}^{2}{\left(\frac{\partial f_{2}}{\partial y}\right)}_{y=\;-1}D}{D_{5}\sin h\left(2A\right)} & D_{5}=\frac{1}{\left(D^{2}-{4\lambda }^{2}\right)\left(D^{2}-\left({4\lambda }^{2}+{\mathrm{iRe}}_{\omega }\right)\right)} \end{array}$$

The correlation coefficient satisfies the following equation $\chi_{j}\gamma_{j}=\;{Г}_{j}$, (j = 3, 4, 5):

(A24) $$\gamma_{3}=\left(\begin{array}{c} c_{33}\\ c_{34} \end{array}\right)$$

(A25) $$\chi_{3}=\left(\begin{array}{cc} \sqrt{{\mathrm{iRe}}_{\omega }}\exp(\sqrt{{\mathrm{iRe}}_{\omega }}) & -\sqrt{{\mathrm{iRe}}_{\omega }}\exp(-\sqrt{{\mathrm{iRe}}_{\omega }})\\ \sqrt{{\mathrm{iRe}}_{\omega }}\exp(-\sqrt{{\mathrm{iRe}}_{\omega }}) & -\sqrt{{\mathrm{iRe}}_{\omega }}\exp(\sqrt{{\mathrm{iRe}}_{\omega }}) \end{array}\right)$$

(A26) $${Г}_{3}=\left(\begin{array}{c} -\frac{1}{2}{\left(\frac{\partial^{2}g_{2}}{\partial y^{2}}\right)}_{y=1}-A_{3}^{\pm }k\sin h\left(2k\right)\\ \mp \frac{1}{2}{\left(\frac{\partial^{2}g_{2}}{\partial y^{2}}\right)}_{y=-1}-B_{3}^{\pm }k\sin h\left(2k\right) \end{array}\right)$$

(A27) $$\gamma_{4}=\left(\begin{array}{c} \gamma_{41}\\ \gamma_{42}\\ \gamma_{43}\\ \gamma_{44} \end{array}\right)$$

(A28) $$\chi_{4}=\left(\begin{array}{cccc} \lambda \exp(\lambda ) & -\lambda \exp(-\lambda ) & C\exp(C) & -C\exp(-C)\\ \lambda \exp(-\lambda ) & -\lambda \exp(\lambda ) & C\exp(-C) & -C\exp(C)\\ \exp(\lambda ) & \exp(-\lambda ) & \exp(C) & \exp(-C)\\ \exp(-\lambda ) & \exp(\lambda ) & \exp(-C) & -\exp(C) \end{array}\right)$$

(A29) $$\Gamma_{4}=\left(\begin{array}{c} -{\left(\frac{\partial^{2}g_{1}}{\partial y^{2}}\right)}_{y=1}-A_{4}^{\pm }A\sin h\left(2A\right)\\ -{\left(\frac{\partial^{2}g_{1}}{\partial y^{2}}\right)}_{y=-1}-B_{4}^{\pm }A\sin h\left(2A\right)\\ -A_{4}^{\pm }\cos h\left(2A\right)-B_{4}^{\pm }\\ -A_{4}^{\pm }-B_{4}^{\pm }\cos h\left(2A\right) \end{array}\right)$$

(A30) $$\gamma_{5}=\left(\begin{array}{c} \gamma_{51}\\ \gamma_{52}\\ \gamma_{53}\\ \gamma_{54} \end{array}\right)$$

(A31) $$\chi_{5}=\left(\begin{array}{cccc} 2\lambda \exp(2\lambda ) & -2\lambda \exp(-2\lambda ) & E\exp(E) & -E\exp(-E)\\ 2\lambda \exp(-2\lambda ) & -2\lambda \exp(2\lambda ) & E\exp(-E) & -E\exp(E)\\ \exp(2\lambda ) & \exp(-2\lambda ) & \exp(E) & \exp(-E)\\ \exp(-2\lambda ) & \exp(2\lambda ) & \exp(-E) & -\exp(E) \end{array}\right)$$

(A32) $$\Gamma_{5}=\left(\begin{array}{c} \frac{1}{2}{\left(\frac{\partial^{2}g_{2}}{\partial y^{2}}\right)}_{y=1}-A_{5}^{\pm }D\sin h\left(2D\right)\\ {\pm \frac{1}{2}\left(\frac{\partial^{2}g_{2}}{\partial y^{2}}\right)}_{y=-1}+B_{5}^{\pm }D\sin h\left(2D\right)\\ -A_{5}^{\pm }\cos h\left(2D\right){\mp \frac{1}{4}\left(\frac{\partial g_{2}}{\partial y}\right)}_{y=1}-B_{5}^{\pm }\\ -A_{5}^{\pm }-B_{5}^{\pm }\cos h\left(2D\right){\mp \frac{1}{4}\left(\frac{\partial g_{2}}{\partial y}\right)}_{y=-1} \end{array}\right)$$

The second-order equation correlation coefficients as well as constant terms.

(A33) $$f_{6}^{\;\pm }\left(y\right)=\frac{P_{6}\sin h[\kappa (1+y)]+Q_{6}^{\pm }\sin h[\kappa (1\;-\;y)]}{\sin h(2\kappa )}$$

(A34) $$f_{7}^{\;\pm }\left(y\right)=\frac{P_{7}\sin h[A(1+y)]+Q_{7}^{\pm }\sin h[A(1\;-\;y)]}{\sin h(2A)}$$

(A35) $$f_{8}^{\;\pm }\left(y\right)=\frac{P_{8}\sin h[D(1+y)]+Q_{8}^{\pm }\sin h[D(1\;-\;y)]}{\sin h(2D)}$$

(A36) $$f_{9}^{\;\pm }\left(y\right)=\frac{P_{9}\sin h[F(1+y)]+Q_{9}^{\pm }\sin h[F(1\;-\;y)]}{\sin h(2F)}$$

(A37) $$F=\sqrt{(3\lambda {)}^{2}+\kappa^{2}}$$

(A38) $$\begin{array}{ccc} P_{6}=\;-\frac{1}{4}{\left(\frac{\partial^{2}f_{1}}{\partial y^{2}}\right)}_{y=1}+\frac{1}{2}{\left(\frac{\partial f_{4}}{\partial y}\right)}_{y=1} & , & Q_{6}^{\pm }=\mp \frac{1}{4}{\left(\frac{\partial^{2}f_{1}}{\partial y^{2}}\right)}_{y=\;-1}\pm \frac{1}{2}{\left(\frac{\partial f_{4}}{\partial y}\right)}_{y=\;-1} \end{array}$$

(A39) $$P_{7}=\;-{\left(\frac{\partial f_{3}}{\partial y}\right)}_{y=1}+\frac{1}{2}{\left(\frac{\partial f_{5}}{\partial y}\right)}_{y=1}-\frac{3}{8}{\left(\frac{\partial^{2}f_{2}}{\partial y^{2}}\right)}_{y=1}$$

(A40) $$Q_{7}^{\pm }=\mp {\left(\frac{\partial f_{3}}{\partial y}\right)}_{y=\;-1}\pm \frac{1}{2}{\left(\frac{\partial f_{5}}{\partial y}\right)}_{y=\;-1}\mp \frac{3}{8}{\left(\frac{\partial^{2}f_{2}}{\partial y^{2}}\right)}_{y=\;-1}$$

(A41) $$\begin{array}{ccc} P_{8}=\frac{1}{2}{\left(\frac{\partial f_{4}}{\partial y}\right)}_{y=1}+\frac{1}{4}{\left(\frac{\partial^{2}f_{1}}{\partial y^{2}}\right)}_{y=1} & , & Q_{8}^{\pm }=\pm \frac{1}{2}{\left(\frac{\partial f_{4}}{\partial y}\right)}_{y=\;-1}\pm \frac{1}{4}{\left(\frac{\partial^{2}f_{1}}{\partial y^{2}}\right)}_{y=\;-1} \end{array}$$

(A42) $$\begin{array}{ccc} P_{9}=\;-\frac{1}{2}{\left(\frac{\partial f_{5}}{\partial y}\right)}_{y=1}+\frac{1}{8}{\left(\frac{\partial^{2}f_{2}}{\partial y^{2}}\right)}_{y=1} & , & Q_{9}^{\pm }=\mp \frac{1}{2}{\left(\frac{\partial f_{5}}{\partial y}\right)}_{y=\;-1}\pm \frac{1}{8}{\left(\frac{\partial^{2}f_{2}}{\partial y^{2}}\right)}_{y=\;-1} \end{array}$$

(A43) $$g_{6}^{\pm }\left(y\right)=c_{63}e^{\sqrt{{\mathrm{iRe}}_{\omega }}y}+c_{64}e^{-\sqrt{{\mathrm{iRe}}_{\omega }}y}+A_{6}^{\pm }\cos h\left[k\left(1+y\right)\right]+B_{6}^{\pm }\cos h\left[k\left(1\;-\;y\right)\right]$$

(A44) $$g_{7}^{\pm }\left(y\right)=c_{71}e^{\lambda y}+c_{72}e^{-\lambda y}+c_{73}e^{\mathrm{Cy}}{+c}_{74}e^{-\mathrm{Cy}}+A_{7}^{\pm }\cos h\left[A\left(1+y\right)\right]+B_{7}^{\pm }\cos h\left[A\left(1\;-\;y\right)\right]$$

(A45) $$g_{8}^{\pm }\left(y\right)=c_{81}e^{2\lambda y}+c_{82}e^{-2\lambda y}+c_{83}e^{\mathrm{Ey}}{+c}_{84}e^{-\mathrm{Ey}}+A_{8}^{\pm }\cos h\left[D\left(1+y\right)\right]+B_{8}^{\pm }\cos h\left[D\left(1\;-\;y\right)\right]$$

(A46) $$g_{9}^{\pm }\left(y\right)=c_{91}e^{3\lambda y}+c_{92}e^{-3\lambda y}+c_{93}e^{\mathrm{Gy}}{+c}_{94}e^{-\mathrm{Gy}}+A_{9}^{\pm }\cos h\left[F\left(1+y\right)\right]+B_{9}^{\pm }\cos h\left[F\left(1\;-\;y\right)\right]$$

(A47) $$\begin{array}{ccc} A_{6}=\frac{\frac{1}{4}{\left(\frac{\partial^{2}f_{1}}{\partial y^{2}}\right)}_{y=1}+\frac{1}{2}{\left(\frac{{\partial f}_{4}}{\partial y}\right)}_{y=1}}{B\sin h\left(2k\right)} & B_{6}^{\pm }=\frac{\mp \left[\frac{1}{4}{\left(\frac{\partial^{2}f_{1}}{\partial y^{2}}\right)}_{y=\;-1}+\frac{1}{2}{\left(\frac{{\partial f}_{4}}{\partial y}\right)}_{y=\;-1}\right]}{B\sin h\left(2k\right)} & . \end{array}$$

(A48)
$$\begin{array}{ccc} A_{7}=\frac{k^{2}{A\left(\frac{\partial f_{3}}{\partial y}-\frac{1}{2}\frac{{\partial f}_{5}}{\partial y}+\frac{3}{8}\frac{\partial^{2}f_{2}}{\partial y^{2}}\right)}_{y=1}}{D_{2}\sin h\left(2A\right)} & B_{7}^{\pm }=\frac{k^{2}A{\left(\mp \frac{\partial f_{3}}{\partial y}\pm \frac{1}{2}\frac{{\partial f}_{5}}{\partial y}\mp \frac{3}{8}\frac{\partial^{2}f_{2}}{\partial y^{2}}\right)}_{y=\;-1}}{D_{2}\sin h\left(2A\right)} & . \end{array}$$

(A49)
$$\begin{array}{ccc} A_{8}=\frac{-k^{2}D{\left(\frac{1}{2}\frac{\partial f_{4}}{\partial y}+\frac{1}{4}\frac{\partial^{2}f_{1}}{{\partial y}^{2}}\right)}_{y=1}}{D_{5}\sin h\left(2A\right)} & B_{8}^{\pm }=\frac{k^{2}D{\left(\pm \frac{1}{2}\frac{\partial f_{4}}{\partial y}\pm \frac{1}{4}\frac{\partial^{2}f_{1}}{{\partial y}^{2}}\right)}_{y=\;-1}}{D_{5}\sin h\left(2A\right)} & . \end{array}$$

(A50)
$$\begin{array}{ccc} A_{9}=\frac{k^{2}F{\left(\frac{1}{2}\frac{\partial f_{5}}{\partial y}-\frac{1}{8}\frac{\partial^{2}f_{2}}{{\partial y}^{2}}\right)}_{y=1}}{D_{9}\sin h\left(2A\right)} & B_{9}^{\pm }=\frac{k^{2}F{\left(\mp \frac{1}{2}\frac{\partial f_{5}}{\partial y}\pm \frac{1}{8}\frac{\partial^{2}f_{2}}{{\partial y}^{2}}\right)}_{y=1}}{D_{9}\sin h\left(2A\right)} & . \end{array}$$

(A51)
$$\begin{array}{cc} D_{9}=\frac{1}{\left(F^{2}-{9\lambda }^{2}\right)\left(F^{2}-\left({9\lambda }^{2}+{\mathrm{iRe}}_{\omega }\right)\right)} & G= \end{array}\sqrt{{9\lambda }^{2}+{\mathrm{iRe}}_{\omega }}$$

The correlation coefficient satisfies the following equation $\chi_{k}\gamma_{k}=\;Гk,$ (j = 6, 7, 8, 9):

(A52) $$\gamma_{6}=\left(\begin{array}{c} c_{63}\\ c_{64} \end{array}\right)$$

(A53) $$\chi_{6}=\left(\begin{array}{cc} \sqrt{{\mathrm{iRe}}_{\omega }}\exp(\sqrt{{\mathrm{iRe}}_{\omega }}) & -\sqrt{{\mathrm{iRe}}_{\omega }}\exp(-\sqrt{{\mathrm{iRe}}_{\omega }})\\ \sqrt{{\mathrm{iRe}}_{\omega }}\exp(-\sqrt{{\mathrm{iRe}}_{\omega }}) & -\sqrt{{\mathrm{iRe}}_{\omega }}\exp(\sqrt{{\mathrm{iRe}}_{\omega }}) \end{array}\right)$$

(A54) $$\Gamma_{6}=\left(\begin{array}{c} {\left[\frac{1}{4}\frac{\partial^{3}g_{1}}{\partial y^{3}}+\frac{1}{2}\frac{\partial^{2}g_{4}}{\partial y^{2}}\right]}_{y=1}-A_{6}^{.}ksinh\left(2k\right)\\ {\mp \left[\frac{1}{4}\frac{\partial^{3}g_{1}}{\partial y^{3}}+\frac{1}{2}\frac{\partial^{2}g_{4}}{\partial y^{2}}\right]}_{y=-1}-B_{6}^{\pm }ksinh\left(2k\right) \end{array}\right)$$

(A55) $$\gamma_{7}=\left(\begin{array}{c} \gamma_{71}\\ \gamma_{72}\\ \gamma_{73}\\ \gamma_{74} \end{array}\right)$$

(A56) $$\chi_{7}=\left(\begin{array}{cccc} \lambda \exp(\lambda ) & -\lambda \exp(-\lambda ) & C\exp(C) & -C\exp(-C)\\ \lambda \exp(-\lambda ) & -\lambda \exp(\lambda ) & C\exp(-C) & -C\exp(C)\\ \exp(\lambda ) & \exp(-\lambda ) & \exp(C) & \exp(-C)\\ \exp(-\lambda ) & \exp(\lambda ) & \exp(-C) & -\exp(C) \end{array}\right)$$

(A57) $$\Gamma_{7}=\left(\begin{array}{c} -{\left(\frac{\partial^{2}g_{3}}{\partial y^{2}}-\frac{1}{2}\frac{\partial^{2}g_{5}}{{\partial y}^{2}}+\frac{3}{8}\frac{\partial^{3}g_{2}}{{\partial y}^{3}}\right)}_{y=1}-A_{7}^{\pm }A\sin h\left(2A\right)\\ \mp {\left(\frac{\partial^{2}g_{3}}{\partial y^{2}}\mp \frac{1}{2}\frac{\partial^{2}g_{5}}{{\partial y}^{2}}\pm \frac{3}{8}\frac{\partial^{3}g_{2}}{{\partial y}^{3}}\right)}_{y=1}+B_{7}^{\pm }A\sin h\left(2A\right)\\ {\left(-\frac{{\partial g}_{5}}{\partial y}+\frac{1}{8}\frac{\partial^{2}g_{2}}{{\partial y}^{2}}\right)}_{y=1}-A_{7}^{\pm }\cos h\left(2A\right)-B_{7}^{\pm }\\ {\left(\mp \frac{{\partial g}_{5}}{\partial y}\pm \frac{1}{8}\frac{\partial^{2}g_{2}}{{\partial y}^{2}}\right)}_{y=-1}-A_{7}^{\pm }-B_{7}^{\pm }\cos h\left(2A\right) \end{array}\right)$$

(A58) $$\gamma_{8}=\left(\begin{array}{c} \gamma_{81}\\ \gamma_{82}\\ \gamma_{83}\\ \gamma_{84} \end{array}\right)$$

(A59) $$\chi_{8}=\left(\begin{array}{cccc} 2\lambda \exp(2\lambda ) & -2\lambda \exp(-2\lambda ) & E\exp(E) & -E\exp(-E)\\ 2\lambda \exp(-2\lambda ) & -2\lambda \exp(2\lambda ) & E\exp(-E) & -E\exp(E)\\ \exp(2\lambda ) & \exp(-2\lambda ) & \exp(E) & \exp(-E)\\ \exp(-2\lambda ) & \exp(2\lambda ) & \exp(-E) & -\exp(E) \end{array}\right)$$

(A60) $$\Gamma_{8}=\left(\begin{array}{c} {\left(\frac{1}{2}\frac{\partial^{2}g_{4}}{\partial y^{2}}+\frac{1}{4}\frac{\partial^{3}g_{1}}{{\partial y}^{3}}\right)}_{y=1}-A_{8}^{\pm }D\sin h\left(2D\right)\\ {\pm \left(\frac{1}{2}\frac{\partial^{2}g_{4}}{\partial y^{2}}+\frac{1}{4}\frac{\partial^{3}g_{1}}{{\partial y}^{3}}\right)}_{y=-1}+B_{8}^{\pm }D\sin h\left(2D\right)\\ -A_{8}^{\pm }\cos h\left(2D\right){-\frac{1}{4}\left(\frac{\partial g_{4}}{\partial y}\right)}_{y=1}-B_{8}^{\pm }\\ -A_{8}^{\pm }-B_{8}^{\pm }\cos h\left(2D\right){\mp \frac{1}{4}\left(\frac{\partial g_{4}}{\partial y}\right)}_{y=-1} \end{array}\right)$$

(A61) $$\gamma_{9}=\left(\begin{array}{c} \gamma_{91}\\ \gamma_{92}\\ \gamma_{93}\\ \gamma_{94} \end{array}\right)$$

(A62) $$\chi_{9}=\left(\begin{array}{cccc} 3\lambda \exp(3\lambda ) & -3\lambda \exp(-3\lambda ) & G\exp(G) & -G\exp(-G)\\ 3\lambda \exp(-3\lambda ) & -3\lambda \exp(3\lambda ) & G\exp(-G) & -G\exp(G)\\ \exp(3\lambda ) & \exp(-3\lambda ) & \exp(G) & \exp(-G)\\ \exp(-3\lambda ) & \exp(3\lambda ) & \exp(-G) & -\exp(G) \end{array}\right)$$

(A63) $$\Gamma_{9}=\left(\begin{array}{c} {\left(-\frac{1}{2}\frac{\partial^{2}g_{5}}{\partial y^{2}}+\frac{1}{8}\frac{\partial^{3}g_{2}}{{\partial y}^{3}}\right)}_{y=1}-A_{9}^{\pm }F\sin h\left(2F\right)\\ {\left(\mp \frac{1}{2}\frac{\partial^{2}g_{5}}{\partial y^{2}}\pm \frac{1}{8}\frac{\partial^{3}g_{2}}{{\partial y}^{3}}\right)}_{y=1}+B_{9}^{\pm }F\sin h\left(2F\right)\\ -A_{9}^{\pm }\cos h\left(2F\right)+{\left(\frac{1}{3}\frac{\partial g_{5}}{\partial y}-\frac{1}{24}\frac{\partial^{2}g_{2}}{{\partial y}^{2}}\right)}_{y=1}-B_{9}^{\pm }\\ -A_{9}^{\pm }-B_{9}^{\pm }\cos h\left(2F\right){+\left(\pm \frac{1}{3}\frac{\partial g_{5}}{\partial y}\mp \frac{1}{24}\frac{\partial^{2}g_{2}}{{\partial y}^{2}}\right)}_{y=\;-1} \end{array}\right)$$

## Appendix B. Numerical Algorithm

Utilizing the finite difference method solves the difference Equations (52)–(54). Since the origin of coordinates is taken at one end of the channel, the values of x and y are in the range of [0, 6] and [−1, 1]. Consider $x_{i}$ (0 ≤ *i* ≤ *n*) and $y_{j}$ (0 ≤ *j* ≤ *m*) as discrete grid points. The steps *τ*$=\frac{T}{k}$, $h_{x}=\frac{L}{N}$, and $h_{y}=\frac{2H}{m}$ divide the x and y axes into n and m components. The dimensionless concentration C of a point ($x_{i}$, $y_{j}$) is denoted as $C_{i,j}$.

The finite difference scheme is used in this paper as the second-order precision in space and the first-order precision in time; that is, O(τ + h2). The time step dt = 0.0005, the space step *dx* = *dy* = 0.1, and the Péclet number Pe varies from 0.3 to 1. The stability condition of the finite difference scheme at this time is as follows:

(A64) $$\frac{({\mathrm{dx}}^{2}+{\mathrm{dy}}^{2})\cdot \mathrm{dt}}{\mathrm{Pe}\cdot {\mathrm{dx}}^{2}\cdot {\mathrm{dy}}^{2}}<\frac{1}{2}$$

The central difference scheme is as follows [40]:

(A65) $$\begin{array}{cc} \frac{\partial C}{\partial x}=\frac{C_{i+1,j}^{n}\;-\;C_{i\;-\;1,j}^{n}}{2h_{x}} & \begin{array}{cc} , & \frac{\partial C}{\partial y}=\frac{C_{i,j+1}^{n}\;-\;C_{i,j\;-\;1}^{n}}{2h_{y}} \end{array} \end{array},$$

(A66) $$\begin{array}{ccc} \frac{\partial^{2}C}{{\partial x}^{2}}=\frac{C_{i+1,j}^{n}\;-\;{2C}_{i,\;j}^{n}+C_{i\;-\;1,j}^{n}}{{h_{x}}^{2}} & , & \frac{\partial^{2}C}{{\partial y}^{2}}=\frac{C_{i,j+1}^{n}\;-\;2C_{i,\;j}^{n}+C_{i,j\;-\;1}^{n}}{{h_{y}}^{2}} \end{array},$$

(A67) $$\frac{\partial C}{\partial t}=\frac{C_{i,j}^{n+1}\;-\;C_{i,j}^{n}}{\tau },$$

(A68) $$\frac{\partial^{3}C}{{\partial y}^{2}}=\frac{C_{i,j+2}^{n}\;-\;{2C}_{i,\;j+1}^{n}+{2C}_{i,\;j\;-\;1}^{n}+C_{i,j\;-\;2}^{n}}{{h_{y}}^{2}}.$$

Then, the Equations (52)–(54) are discretized as follows:

(A69) $$\begin{array}{c} \delta^{0}:\\ C_{0(i,j)}^{n+1}=C_{0(i,\;j)}^{n}\;-\;\frac{\tau }{2h_{x}}{U_{0\left(i,\;j\right)}^{n}(C}_{0\left(i+1,j\right)}^{n}\;-\;C_{0\left(i\;-\;1,j\right)}^{n})\;-\;\frac{\tau }{2h_{y}}V_{0\left(i,\;j\right)}^{n}{(C}_{0\left(i,j+1\right)}^{n}\;-\;C_{0\left(i,j\;-\;1\right)}^{n})+\frac{\tau }{{Peh_{x}}^{2}}{(C}_{0(i+1,j)}^{n}\;-\;{2C}_{0(i,j)}^{n}+C_{0(i\;-\;1,j)}^{n})+\\ \frac{\tau }{{Peh_{y}}^{2}}{(C}_{0(i,j+1)}^{n}\;-\;{2C}_{0(i,j)}^{n}+C_{0(i,j\;-\;1)}^{n}) \end{array}$$

(A70) $$\begin{array}{c} \delta^{1}:\\ C_{1(i,j)}^{n+1}=C_{1(i,\;j)}^{n}\;-\;\frac{\tau }{2h_{x}}{U_{0\left(i,\;j\right)}^{n}(C}_{1\left(i+1,j\right)}^{n}\;-\;C_{1\left(i\;-\;1,j\right)}^{n})\;-\;\frac{\tau }{2h_{x}}{U_{1\left(i,\;j\right)}^{n}(C}_{0\left(i+1,j\right)}^{n}\;-\;C_{0\left(i\;-\;1,j\right)}^{n})\;-\;\frac{\tau }{2h_{y}}V_{0\left(i,\;j\right)}^{n}{(C}_{1\left(i,j+1\right)}^{n}\;-\;C_{1\left(i,j\;-\;1\right)}^{n})\;-\;\\ \frac{\tau }{2h_{y}}V_{1\left(i,\;j\right)}^{n}{(C}_{0\left(i,j+1\right)}^{n}\;-\;C_{0\left(i,j\;-\;1\right)}^{n})+\frac{\tau }{{Peh_{x}}^{2}}{(C}_{1(i+1,j)}^{n}\;-\;{2C}_{1(i,j)}^{n}+C_{1(i\;-\;1,j)}^{n})+\frac{\tau }{{Peh_{y}}^{2}}{(C}_{1(i,j+1)}^{n}\;-\;{2C}_{1(i,j)}^{n}+C_{1(i,j\;-\;1)}^{n}) \end{array}$$

(A71)
$$\begin{array}{c} \delta^{2}:\\ C_{1(i,j)}^{n+1}=C_{1(i,\;j)}^{n}\;-\;\frac{\tau }{2h_{x}}{U_{0\left(i,\;j\right)}^{n}(C}_{1\left(i+1,j\right)}^{n}\;-\;C_{1\left(i\;-\;1,j\right)}^{n})\;-\;\frac{\tau }{2h_{x}}{U_{1\left(i,\;j\right)}^{n}(C}_{0\left(i+1,j\right)}^{n}\;-\;C_{0\left(i\;-\;1,j\right)}^{n})\;-\;\frac{\tau }{2h_{y}}V_{0\left(i,\;j\right)}^{n}{(C}_{1\left(i,j+1\right)}^{n}\;-\;C_{1\left(i,j\;-\;1\right)}^{n})\;-\;\\ \frac{\tau }{2h_{y}}V_{1\left(i,\;j\right)}^{n}{(C}_{0\left(i,j+1\right)}^{n}\;-\;C_{0\left(i,j\;-\;1\right)}^{n})+\frac{\tau }{{Peh_{x}}^{2}}{(C}_{1(i+1,j)}^{n}\;-\;{2C}_{1(i,j)}^{n}+C_{1(i\;-\;1,j)}^{n})+\frac{\tau }{{Peh_{y}}^{2}}{(C}_{1(i,j+1)}^{n}\;-\;{2C}_{1(i,j)}^{n}+C_{1(i,j\;-\;1)}^{n}) \end{array}$$

And the boundary condition:

(A72) $$\begin{array}{c} \delta^{0}:\\ C_{0(0,\;j)}^{n}=1,\;C_{0(n,\;j)}^{n}=0\\ C_{0(i,-1)}^{n}=C_{0(i,1)}^{n},\;j=0\\ C_{0(i,m-1)}^{n}=C_{0(i,m+1)}^{n},\;j=m \end{array}$$

(A73)
$$\begin{array}{c} \delta^{1}:\\ C_{1(0,\;j)}^{n}=0,\;C_{1(n,\;j)}^{n}=0\\ C_{1(i,-1)}^{n}=C_{1(i,1)}^{n}-\sin(\lambda x_{i})\frac{C_{0(i,\;1)}^{n}-{2C}_{0(i,\;0)}^{n}+C_{0(i,\;-1)}^{n}}{{h_{y}}^{2}},\;j=0\\ C_{1(i,m+1)}^{n}=C_{1(i,m-1)}^{n}\mp \sin(\lambda x_{i})\frac{C_{0(i,\;m+1)}^{n}-{2C}_{0(i,\;m)}^{n}+C_{0(i,\;m-1)}^{n}}{{h_{y}}^{2}},\;j=m \end{array}$$

(A74)
$$\begin{array}{c} \delta^{2}:\\ C_{2(0,\;j)}^{n}=0,\;C_{2(n,\;j)}^{n}=0\\ C_{1(i,-1)}^{n}=C_{1(i,1)}^{n}-\sin\left(\lambda x_{i}\right)\frac{C_{1(i,\;1)}^{n}-{2C}_{1(i,\;0)}^{n}+C_{1(i,\;-1)}^{n}}{{h_{y}}^{2}}-\frac{1}{2}\mathrm{si}n^{2}\left(\lambda x_{i}\right)\frac{C_{0(i,2)}^{n}-{2C}_{0(i,\;1)}^{n}+{2C}_{0(i,-1)}^{n}+C_{0(i,-2)}^{n}}{{h_{x}}^{2}},\;j=0\\ C_{1(i,m+1)}^{n}=C_{1(i,m-1)}^{n}\mp \sin\left(\lambda x_{i}\right)\frac{C_{1(i,\;m+1)}^{n}-{2C}_{1(i,\;m)}^{n}+C_{1(i,\;m-1)}^{n}}{{h_{y}}^{2}}\\ \mp \frac{1}{2}\mathrm{si}n^{2}\left(\lambda x_{i}\right)\frac{C_{0(i,m+2)}^{n}-{2C}_{0(i,\;m+1)}^{n}+{2C}_{0(i,m-1)}^{n}+C_{0(i,m-2)}^{n}}{{h_{x}}^{2}},\;\;j=m \end{array}$$
